# Bioinformatic analysis of the CLE signaling peptide family

**DOI:** 10.1186/1471-2229-8-1

**Published:** 2008-01-03

**Authors:** Karsten Oelkers, Nicolas Goffard, Georg F Weiller, Peter M Gresshoff, Ulrike Mathesius, Tancred Frickey

**Affiliations:** 1 School of Biochemistry and Molecular Biology, The Australian National University, Canberra, ACT, Australia; 2 Research School of Biological Sciences, The Australian National University, Canberra, ACT, Australia; 3 The University of Queensland, Brisbane, QLD, Australia; 4 The Australian Research Council Centre of Excellence for Integrative Legume Research

## Abstract

**Background:**

Plants encode a large number of leucine-rich repeat receptor-like kinases. Legumes encode several LRR-RLK linked to the process of root nodule formation, the ligands of which are unknown. To identify ligands for these receptors, we used a combination of profile hidden Markov models and position-specific iterative BLAST, allowing us to detect new members of the CLV3/ESR (CLE) protein family from publicly available sequence databases.

**Results:**

We identified 114 new members of the CLE protein family from various plant species, as well as five protein sequences containing multiple CLE domains. We were able to cluster the CLE domain proteins into 13 distinct groups based on their pairwise similarities in the primary CLE motif. In addition, we identified secondary motifs that coincide with our sequence clusters. The groupings based on the CLE motifs correlate with known biological functions of CLE signaling peptides and are analogous to groupings based on phylogenetic analysis and ectopic overexpression studies. We tested the biological function of two of the predicted CLE signaling peptides in the legume *Medicago truncatula*. These peptides inhibit the activity of the root apical and lateral root meristems in a manner consistent with our functional predictions based on other CLE signaling peptides clustering in the same groups.

**Conclusion:**

Our analysis provides an identification and classification of a large number of novel potential CLE signaling peptides. The additional motifs we found could lead to future discovery of recognition sites for processing peptidases as well as predictions for receptor binding specificity.

## Background

Genomes of higher plants contain a large number of receptor-like kinases (RLK) [1,2]. Leucine-rich repeat RLK (LRR-RLK) form the largest subfamily within plant RLK and mediate protein-protein interactions [3,4]. A group of potential receptor ligands for LRR-RLK are CLV3/ESR (CLE) signaling peptides, first described by Cock and McCormick [5], and recently reviewed [6-8]. Altogether, 65 CLE members are known from a variety of monocotyledonous and dicotyledonous plants. The single CLE signaling peptide known to be present in a non-plant species is encoded by the plant parasitic nematode *Heterodera glycines *[9], and it has been proposed that the parasite acquired the plant signal to alter its host's behavior [10,11]. Apart from this single exception, it has been suggested that CLE signaling peptides are plant-specific [5,12].

Cock and McCormick [5] reported a CLV3-like gene family, that they identified using iterative searches with position-specific iterative BLAST (PSI-BLAST). The authors were able to detect 42 sequences from genomic and expressed sequence tag (EST) databases, yielding 39 related protein sequences. The protein family was termed CLV3/ESR-related (CLE) and is characterized by a conserved domain at the C-terminus spanning 12 residues and a hydrophobic signal peptide at the N-terminus. The variable region (N-terminal relative to the CLE motif) of the protein is thought to have no specific function, as it can be substituted with nucleotides from other genes [13].

The first identified CLE members were termed ESR genes as they were shown to be specifically expressed in the embryo surrounding region (ESR) of *Zea mays *endosperm [14] and their mRNA constitutes the major proportion of the mRNA in the ESR region [15]. The best described member of the CLE family is CLAVATA 3 (CLV3) which is presumed to be the ligand of a CLV1/CLV2 receptor complex. The receptor complex is required for limiting the number of stem cells at the shoot apical meristem (SAM) and forms the paradigm of plant LRR-RLK signaling. A variety of analyses suggest that CLV3 is the ligand perceived by a CLV1/CLV2 receptor heterodimer [16-19]. However, direct binding of the ligand to the receptor has not yet been shown. Overexpression of CLV3 in *Arabidopsis thaliana *hampers the initiation of organs at the SAM after emergence of the first leaves. In *clv3 *loss-of-function mutants, stem cells accumulate at the centre of shoot and floral meristems, additional organs or undifferentiated tissue are formed [17].

Functional characterization of CLE members showed them to be involved in a variety of developmental mechanisms in plants, such as the SAM, the root apical meristem (RAM) or vascular cell differentiation [10,13,20-26]. The exact function of individual CLE signaling peptides remains, however, largely unknown. Analyses in *A. thaliana *showed similar phenotypes after ectopic expression of 18 different CLE signaling peptides and resulted in the classification of CLE members into four groups according to their overexpression phenotypes. This classification correlates with sequence characteristics of the conserved domain [12]. However, the *in vivo *function of the peptides might lead to more specific phenotypes, as their expression pattern in the plant might be local, and not correlate with the ectopic application of active peptides as performed in the assays.

In legumes, the formation of root nodules is triggered by nitrogen fixing bacteria generically called rhizobia [27]. Rhizobia induce new meristems inside the legume root. This process involves at least two known LRR-RLKs. At the early stages of infection, an LRR-RLK, named NORK (NOdulation Receptor Kinase, *Medicago sativa*) [28], DMI2 (Doesn't Make Infections 2, *M. truncatula*) [28], SYMRK (SYMbiosis Receptor Kinase, *Lotus japonicus*) [29], or SYM19 (SYMbiosis 19, *Pisum sativum*) [30] perceives a so far unknown ligand which then activates a signaling cascade leading to nodulation. The proliferation of nodule meristems is limited by the plant. This process, so-called autoregulation of nodulation, is under control of the CLV1-like LRR-RLK NARK (Nodulation Autoregulation Receptor Kinase, *Glycine max*) [31], HAR1 (Hypernodulation Aberrant Root 1, *L. japonicus*) [32], SUNN (SUperNumerary Nodules, *M. truncatula*) [33], and SYM29 (SYMbiosis 29, *P. sativum*) [34]. In all four of these legume species, loss-of-function mutations in this protein result in an uncontrolled proliferation of nodule meristems. The regulation of nodulation is also linked to the nitrogen supply of the plant. If enough nitrogen is available in the soil, nodulation is suppressed [35]. Interestingly, CLE signaling peptides could be involved in the response of plants to nitrogen as an altered expression of CLE2 in *A. thaliana *was observed under nitrogen deprivation [36].

Several authors suggest that a CLE signaling peptide could act as ligand for the autoregulation of nodulation receptor kinase in legumes [21,37]. It is therefore conceivable that CLE domain proteins may play a crucial role in nodule meristem initiation and/or maintenance. So far, only seven CLE members from legumes have been identified. Their role remains unknown. To characterize CLE domain proteins functionally and to test for an involvement in the repression of root nodule meristem formation it is necessary to identify more members from this family. Because of the limited number of known CLE domain proteins from legumes, we systematically surveyed CLE sequences in a large number of plant sequence databases. We analyzed sequences of legumes against a background of known and new non-legume CLE sequences to find out whether any legume-specific CLE domain proteins could be identified.

Due to their size, many small proteins, including potential signaling peptides, are frequently not detected by automated annotation programs. More refined bioinformatics approaches are therefore necessary to identify potential plant signaling peptides, either at the protein or nucleotide level [5,38-42]. In regards to the CLE family, the majority of members were identified using PSI-BLAST and relying on sequence similarity to known CLE members [5,43]. MEME/MAST, a motif detection and search tool, was used to search for CLE sequences in *H. glycines *[9,44]. Several studies also used BLAST for the identification of a limited number of CLE signaling peptides [12,26,45].

## Results

The approach we used for the identification of CLE domain proteins is analogous to the one used in the first report of the CLE family [5]. However, our approach relied on identification of potential CLE family members using a novel combination of PSI-BLAST and HMMer [43,46,47]. PSI-BLAST was used instead of BLAST to detect potential sequence homologues, as PSI-BLAST combines the speed of BLAST with a higher sensitivity, by taking the results of former searches into account and adapting the scoring matrix for subsequent searches. This allows the scoring matrix to better reflect the protein family being analyzed and allows detection of remote members of the sequence family that simple pairwise comparison would fail to detect. HMMer, on the other hand, generates a profile hidden Markov model (HMM) of a sequence family based on a multiple sequence alignment. Given that a high-quality sequence alignment is used, this can provide an even better representation of the sequence family and allow more distant family members to be identified. The downside is that HMMer searches against large sequence databases are quite time consuming. To utilize the best of both approaches we used HMMaccel [48], a program combining PSI-BLAST with HMMer. PSI-BLAST is used in a first step to reduce a large sequence database to a smaller set of sequences showing a minimal amount of sequence similarity to the protein family of interest. In this case, the reduced database consisted of those sequences generating high scoring sequence pairs up to E-values of 10,000. This smaller set of sequences can then be searched using the slower but more exact HMM approach. Thanks to an increased knowledge of CLE domain proteins we could use the previously identified additional sequence characteristics, N-terminal signal sequence and C-terminal conserved domain, as further criteria for assigning motif containing protein sequences to the family.

### Identification of CLE signaling peptides

A custom database using sequence resources from a variety of plant species was generated. We combined sequence data from genome projects for *M. truncatula*, *Oryza sativa*, *Populus trichocarpa *and *A. thaliana*, as well as ESTs from the TIGR Gene Indices [49], and TIGR Plant Transcript Assemblies [50] from legume species and various plants. This yielded a database containing data from a variety of sequencing projects and incorporating a maximum of sequence information, albeit in a redundant form. We included the moss *Physcomitrella patens *and the green alga *Chlamydomonas reinhardtii*, to infer the evolutionary origin of the CLE protein family. The primary input for the iterative search using HMMaccel consisted of a multiple sequence alignment of 45 of the CLE sequences known at the start of the project. A sequence alignment was generated using ClustalW [51] and manually refined. This alignment served as input for HMMaccel, which was used to iteratively search the above mentioned plant databases with a combination of PSI-BLAST and HMMer to detect further homologs. Iteration one produced 169 candidates, iteration two 227 and iteration three 811. Examination of iteration three showed that many sequences were being detected that, while showing some sequence similarity to the known CLE sequences, did not adequately represent the conserved 12 amino acids at the C-terminus. This indicated our HMM having reached the limits of what could be reliably detected based solely on the sequence conservation in this family. To reduce the number of false-positives in the dataset, we analyzed the 811 candidate CLE sequences in CLANS [52,53]. All sequences that did not connect to the central cluster containing the known CLE sequences at a P-value threshold of 1E-04 were removed from the dataset. This threshold was chosen, as none of the excluded sequences contained the 12 amino acids of the CLE motif, whereas increasing the threshold to 1E-05 excluded valid representatives from the dataset. Having refocused the set of sequences to what we believed to be true-positive hits, the remaining 499 sequences were used to seed a fourth iteration of the HMMaccel search. The aim of this search was to detect all true CLE representatives rather than generating a set of sequences containing only true hits and no false-positives. This final iteration also served to recover any true positive sequences we may have inadvertently discarded in the CLANS filtering procedure or that were missed in the third iteration due to a degeneration of the HMM. Iteration four returned 659 sequences. The fact that less sequences were found in iteration four than in iteration three, although more sequences were used to seed the search in iteration four, points to iteration three having returned many true-positive as well as some false-positive sequences and the subsequent CLANS filtering having succeeded in excluding most of the false-positive hits and refocusing the search on true CLE sequences. Iteration four concluded our search for putative CLE signaling peptide sequences.

As a control, we determined whether 20 recently identified members of the family, that had not been included in the initial set of 45 sequences, but had been present in the database, were correctly identified in iteration four. All 20 sequences could be found in the final dataset. Starting from the initial 45 sequences, we also tested whether any of the sequences from previous iterations were lost in subsequent iterations, which would indicate a drift of the dataset. This was performed for the first three iterations but was not applicable for the fourth, as sequences had been manually removed from the dataset. We could not detect a noticeable drift of the dataset as, at most, three sequences were lost between successive iterations. The 45 CLE members, serving as initial seeds for the search performed in iteration one, were consistently recovered throughout the following iterations. The only known CLE sequences we were unable to detect were CLE8 (*A. thaliana*) [5,53] and CLE15 (*O. sativa*) [5], as these were not present in our database. The closest homologues we could identify for CLE8 were other known CLE members with high sequence identity in the conserved CLE domain. We were unable to detect any sequence showing a high degree of similarity to CLE8 over the entire length of the protein. For CLE15, we were able to identify two close homologues (*O. sativa *TIGR EST entries TC281944_+1 and NP936837_+1). A multiple sequence alignment revealed that both EST entries do not contain a CLE motif, but are identical with CLE15 in the remaining sequence. This indicates that the assembly of the EST changed. Therefore, we concluded that the sequences originally identified as CLE8 and CLE15 had been removed from the database version that was used for this study. All other known CLE sequences were identified in the course of this iterative search.

Next, we eliminated false positive candidates from the 659 sequences obtained in the final HMMaccel search. There is no stereotype CLE member in regards to the primary protein sequence and slight variations in the sequence of the CLE motif occur throughout the known family members. Consequently, the tandem repeats described by Strabala et al. [12] and stringent criteria based on the primary sequence were set up to reliably assign candidates to the CLE family. The primary characteristic of the CLE family is the amino acid sequence of the conserved C-terminal region. As second criteria, protein length (60–120 amino acids) and relative position of the motif in the sequence were considered. Commonly, the motif is localized at the C-terminus, well within the last third of the full-length sequence. As a third criterion the isoelectric point was considered, as the vast majority of known CLE sequences have a basic pI. Of the 659 sequences, we eliminated 303 sequences that did not conform to the above criteria leaving 356 potential CLE domain proteins.

Many sequences were represented multiple times with varying identifiers as our custom database was generated by pooling multiple sequence databases together. To reduce the redundancy of our final set, we grouped the 356 sequences by sequence similarity using CD-Hit [54]. CD-Hit clusters were calculated with different thresholds ranging from 70–100% identity. To make the dataset non-redundant, sequences were sorted according to their 70% identity-threshold and all sequences assigned to the same cluster were grouped. Groups containing sequences with less than 99% identity were manually validated using MultAlin [55]. This process resulted in a final set of 179 non-redundant sequences, which included the 65 known and 114 novel CLE domain proteins (Table 1, Additional File 1).

**Table 1:** Known and identified CLE signaling peptides

Species	Overall redundant	New non-redundant	Known non-redundant	Overall non-redundant
*Arabidopsis thaliana*	83	1	31	32
*Brassica napus*	5	2	1	3
*Chlamydomonas reinhardtii*	2	1	0	1
*Glycine max*	43	13	2	15
*Gossypium hirsutum*	ND	ND	1	1
*Heterodera glycines*	1	0	1	1
*Lotus japonicus*	1	1	0	1
*Lycopersicum esculentum*	7	3	1	4
*Medicago truncatula*	31	11	5	16
*Nicotiana tabacum*	2	1	0	1
*Oryza sativa*	89	31	13	44
*Phaseolus coccineus*	1	1	0	1
*Phaseolus vulgaris*	2	2	0	2
*Physcomitrella patens*	2	1	0	1
*Populus trichocarpa*	35	26	0	26
*Solanum tuberosum*	9	5	0	5
*Triticum aestivum*	ND	ND	3	3
*Zea mays*	41	15	6	21
*Zinnia elegans*	ND	ND	1	1

Overview of the identification of potential CLE signaling peptides from plant species with newly identified and known CLE members. ND – not determined in this study.

There is confusion in the nomenclature of the family. We attempted to keep naming of the CLE family members objective and consistent. Similar the approach by Cock and McCormick [5] every member was consecutively numbered and prefixed with "CLE", independent of species origin. We also assigned CLE numbers to those members which had not yet been included in a systematic nomenclature (e.g., CLV3, TDIF, *Hg*CLE, *Bn*CLE19).

Independently, we searched a custom database containing sequences from symbiotic bacteria (*Bradyrhizobium japonicum*, *Sinorhizobium meliloti, Mesorhizobium loti*), pathogenic bacteria (*Agrobacterium tumefaciens, Agrobacterium rhizogenes*), symbiotic fungi (*Glomus interadices, Laccaria bicolor*) and a range of pathogenic fungi (e.g., *Ustilago maydis, Botrytis cinerea, Phytophthora sojae*) to see whether any non-plant CLE sequence could be detected. No CLE candidate sequences could be detected in any of these species.

Finally, we searched the non-redundant protein database from NCBI (nr) using the HMM derived from the filtered results of iteration three. CLE sequences returned by this search were solely from plants, with the single exception of the previously identified CLE member from *H. glycines *[10]. In addition, searching the nr database did not reveal any sequences we had not previously identified using our custom plant database.

### CLE members with multiple and regularly arranged CLE domains

**Figure 1:**
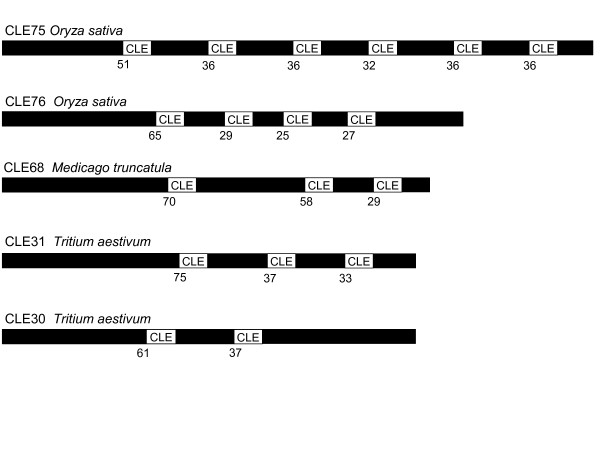
**Multidomain CLE sequences**. The potential multidomain CLE signaling peptides CLE75, CLE76, CLE68, CLE31 and CLE30 are represented. The figure is a scaled representation of the domain organization. The relative positions of the first amino acid of the motifs are specified.

A general characteristic of the CLE family is that members contain a single conserved domain. Surprisingly, we found five sequences (CLE75, CLE76, CLE68, CLE30, CLE31) from three plant species which contained multiple CLE motifs (Table 2). The sequences encoding CLE75 and CLE76 had one entry each in the *O. sativa *genome, originating from two different genomic loci on chromosome 5. CLE68 had one entry in the *M. truncatula *genome. CLE30 and CLE31 from *T. aestivum *were identified by Cock and McCormick and originate from the *T. aestivum *EST database [5]. In all five cases, the conserved CLE motifs within one protein sequence are very similar to one another and carry the same variations within the CLE motif. CLE68 from *M. truncatula *is an exception, as the third domain is different from the first two domains in the protein sequence. In all cases, the CLE domains are regularly arranged, with the first domain occurring after 50–75 amino acids, which is typical for standard CLE members, and further domains occurring at intervals of approximately 30 amino acids (Figure 1). Again, CLE68 from *M. truncatula *forms an exception with a larger gap between the first and the second domain. The sequences positioned in between consecutive CLE motifs are similar to one another, indicating a fusion of tandem duplications of the gene or a mis-annotation of the genome or EST entry.

**Table 2:** Detailed characteristics of multi-CLE domain proteins

CLE	Database	Length	Motif	Start	Stop	Motif Sequence	Distance
CLE75	*O. sativa *genome	250	1	51	63	IGVG**KRLTPTGPNPVHN**EFQP	51
			2	87	99	IGNG**KRLTPTGPDPIHN**EFQP	36
			3	123	135	IGDG**KRLTPTGPDPVHN**KFQP	36
			4	155	167	IGDG**KRLTPTGPDPIHN**EFQP	32
			5	191	203	IGDG**KRLTPIGPDPIHN**EFPP	36
			6	223	235	IGDG**KRLTPTGPDPVHN**EFQP	36
CLE76	*O. sativa *genome	195	1	65	77	DFSV**LRKVPTGPDPITS**DPPP	65
			2	94	106	QFSV**LRKVPTGPDPITS**DPPP	29
			3	119	131	EFPV**LREVPSGPDPITS**DPPP	25
			4	146	158	EFPV**LREVPSGPDPITS**DPPP	27
CLE68	*M. truncatula *genome	181	1	70	82	EIGE**LRKVPSSPDPIHN**SDID	70
			2	128	140	QIRG**LTKVPTSPDPIHN**SDSV	58
			3	157	169	QIGR**ARMVSSGPNPLHN**RLIN	29
CLE31	*T. aestivum *ESTs	175	1	75	97	IMMA**PRPVPSGPDPIHH**CPPA	75
			2	112	124	AMVA**PRPVPSGPNPIHH**RPPH	37
			3	145	157	VMVA**PMPIPSGPDPIHH**CPPA	33
CLE30	*T. aestivum *ESTs	175	1	61	73	VMVA**PRPVPSGPDPIHH**RPHA	61
			2	98	110	VMVA**PRPVPSGPNPIHH**FPAP	37

Detailed characteristics of identified CLE members that carry multiple CLE motifs. The table contains information about database origin, protein length in amino acids, and motif position as well as motif sequences and distance in amino acids between the motifs in the protein sequence.

### Sequence analysis

**Figure 2:**
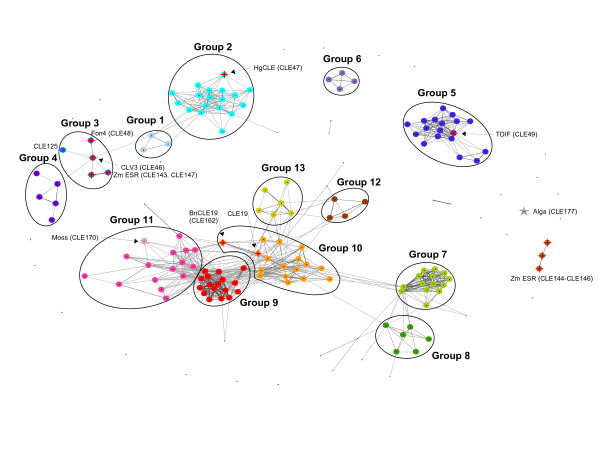
**Analysis of sequence similarity in the CLE domain**. CLANS clustering of 174 sequences based on their sequence similarity in the CLE domain. Sequences are represented by dots and the various groups are highlighted by ovals. Sequences of the same group are assigned the same color. Lines connecting the dots correspond to BLASTP values better than 1.2E-7. Characterized CLE members *Hg*CLE (CLE47), TDIF (CLE49) and *Zm*ESR (CLE143–CLE147), as well as the known orthologs CLV3/FON4 and CLE19/*Bn*CLE19 (CLE162) are highlighted with red stars. The single CLE member found from *Physcomitrella patens *(moss, CLE170), which clusters into group 11, is highlighted with a grey star. A putative CLE sequence from *Chlamydomonas reinhardtii *(alga, CLE177) is also marked with a grey star but does not cluster close to any group. The grouping established upon cluster analysis is analogous to previous classifications [8, 12, 24]. Group 2 contains CLE1–CLE7, which were previously shown to have no effect on RAM growth or on vascular cell differentiation in peptide assays and which led to *wus*-like dwarf growth only at 21 days after germination when ectopically overexpressed. CLE9–CLE13 can be found in group 7. These CLE members had an effect on the RAM but not on vascular cell differentiation in peptide assyas and *wus*-like dwarf growth could be observed at 14 and 21 days after germination in overexpression studies. The CLE family members CLE41, CLE42, CLE44, which had no effect on RAM but on vascular cell differentiation in peptide assays, and had a *shrub*-like overexpression phenotype are located in group 5.

The majority of the overall protein sequence of CLE members appears unrelated; sequence similarity within the family is essentially confined to a conserved domain of 12–18 amino acids at the C-terminus. We carried out detailed sequence analyses, firstly to search for similarity within the CLE motif (12–18 amino acids), and secondly to test whether there is any sequence similarity outside the CLE motif. We performed cluster analyses of the conserved domains of the family using CLANS [52,53]. CLANS is a Java tool to visualize and analyze protein sequence similarity based on pairwise similarity (BLAST) and well suited for the analysis of large sets of sequences. CLANS does not allow drawing phylogenetic conclusions, it solely allows analyzing protein sequence similarity. The clustering of the sequences led to the classification of 136 sequences into 13 groups (Figure 2). We excluded the five CLE members carrying multiple CLE domains from the graph, as these complicated the visualization. 38 sequences, which comprise known as well as newly identified CLE members, could not be reliably assigned to any of the 13 groups.

**Figure 3:**
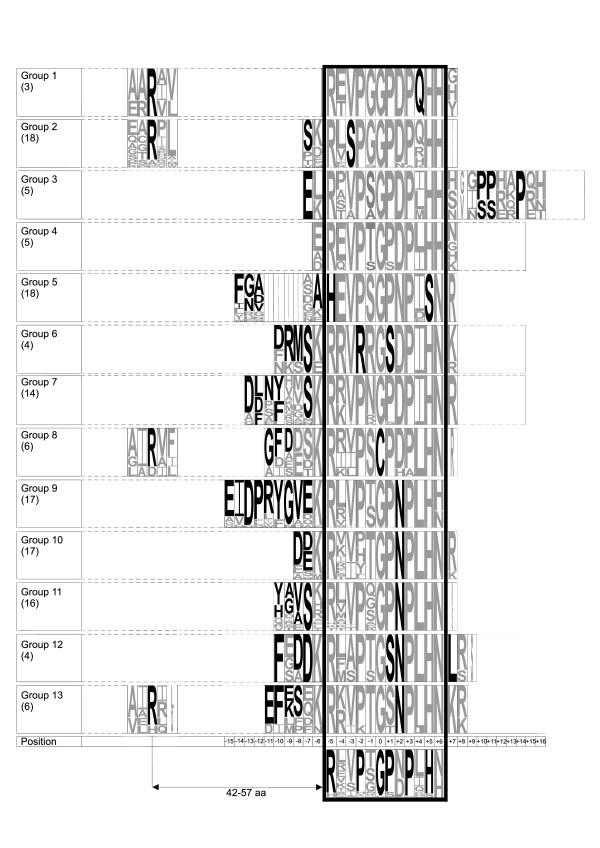
**Weblogo representation of the conservation pattern of residues in each group and for the entire protein family**. The previously described main CLE motif of 12 amino acid length is marked with a black frame. Group specific residues are marked in black in the various groups. Invariant residues are marked in black in the bottommost logo. Conserved residues are marked grey. The size of the letter symbolizes the frequency of that residue in the group and at that position. A secondary motif was identified at around 50 amino acids upstream of the primary CLE motif in groups 1, 2, 8 and 13. Extensions of the motif are recognizable at both the C- and N-terminus. Bracketed figures indicate the number of sequences assigned to the respective group.

After clustering, we analyzed the sequence similarity of the entire protein sequence to see whether the sequences grouped by their CLE motif had similar sequence regions outside the motif. We built sequence logos to visualize conserved residues within and outside the 12 amino acid CLE motif. Within the CLE motif, the sequence consensus over the whole family reveals that there are six residues which are almost invariant (Figure 3). These include R, P, G, P, P and H, of which the first two P residues were found to be hydroxylated [24]. Because of the central conserved position of G, we assigned G to the position zero and numbered the positions of the other amino acids relative to this G. There are two positions which have an equal probability of occurrence for N and D as well as for N and H. These conserved residues might provide a framework for the receptor interaction of the presumed ligands. Some rare variations in these conserved residues occur in position 0 (C instead of G in group 8 only) and position +1 (S instead of the predominant hydroxylated P in groups 6 and 12). Other positions in the domain are rather variable, such as positions -4 and -1. We were able to identify group-specific residues, i.e. residues that are responsible for the separation into distinct groups based on CLANS, which are highlighted in Figure 3.

An analysis of the protein sequence regions adjacent to the CLE motif showed that, rather than being random, certain regions outside the CLE motif were conserved (Figure 3). Interestingly, these conserved motifs followed the groupings based on CLANS. This shows that the sequence of the primary CLE motif correlates with further regions of sequence similarity, possible secondary sequence motifs, in other parts of the coding region of CLE proteins.

### Biological function of identified CLE signaling peptides in *Medicago truncatula*

To confirm the biological activity of the *in silico *identified CLE members we tested synthetic peptides corresponding to the conserved CLE domain in a peptide assay. Since the majority of CLE sequences are predicted to have an effect on the growth of the RAM, we used peptides that we expected to have an effect on the RAM based our grouping (Figure 2). We synthesized two peptides, peptide 1 (SKRKVPSCPDPLHN) and peptide 2 (SKRRVPNGPDPIHN). The length of 14 amino acids was chosen, as such peptides were shown to be active in previous reports [22]. Peptide 1 was only found in one CLE member, CLE67 of *M. truncatula*, which clustered in group 9 (Figure 2, Additional File 2). Peptide 2 was present in a total of eight CLE sequences from various plant species CLE34, CLE36, CLE64, CLE78, CLE80, CLE117, CLE118 and CLE163, due to the redundancy in the conserved domain. Because the CLE domain that was used for clustering included up to 18 amino acids, some of the latter CLE sequences were grouped into different groups, including group 7 (CLE34, CLE78, CLE80, CLE117, CLE118, CLE163), group 8 (CLE64) and one was ungrouped but located close to groups 7 and 8 (CLE36). As a control, we used two peptides with individually randomized sequence (peptide 3 and peptide 4) having the same amino acid composition, molecular weight and isoelectric point as peptide 1 and 2, respectively.

**Figure 4:**
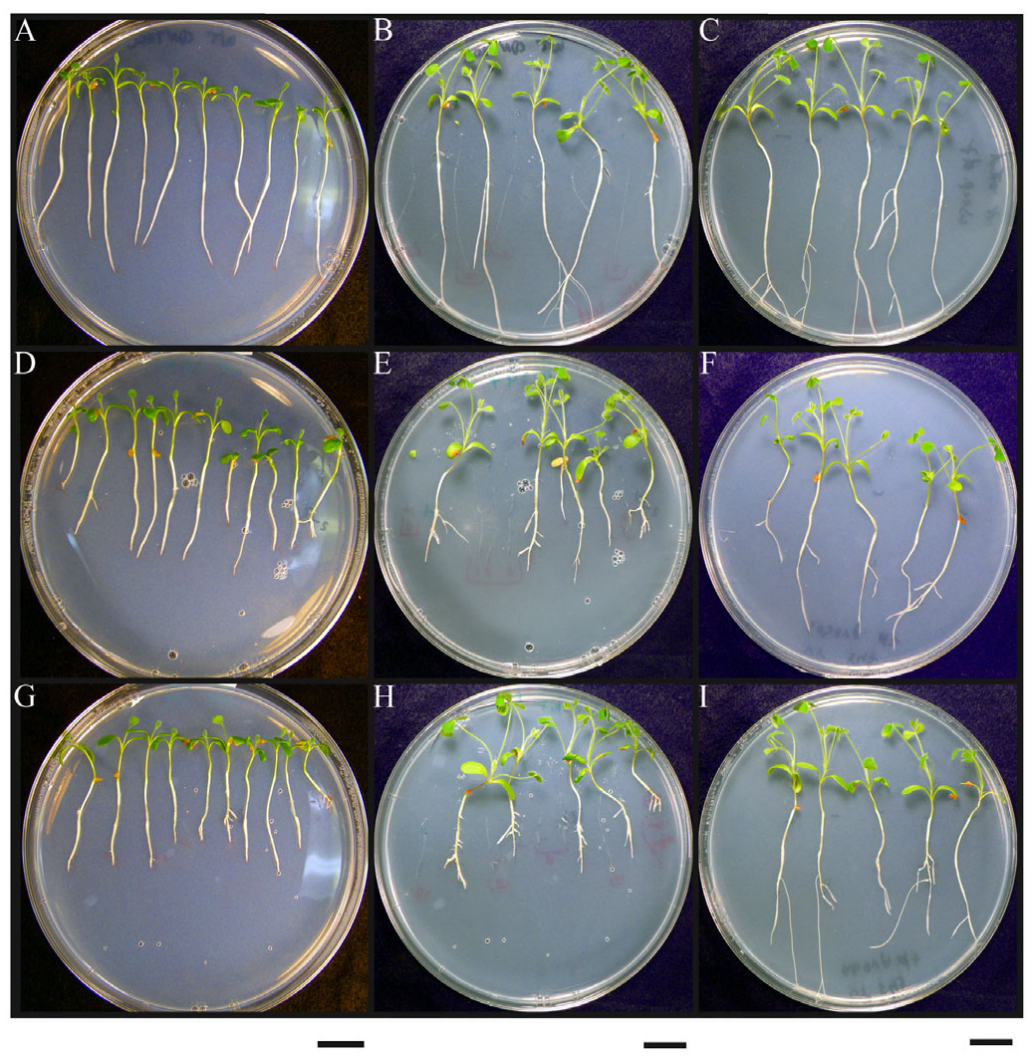
**Biological activity of CLE peptides in *Medicago truncatula***. Confirmation of the biological activity of synthetic CLE peptides corresponding to 14 amino acids of the conserved domain of predicted CLE signaling peptides in a plate assay using *M. truncatula*. Peptides were added at a concentration of 10 μM as growth media additives. The top row (A-C) shows plant growth in the absence of peptide, the middle row (D-F) in the presence of peptide 1 (SKRKVPSCPDPLHN), and the bottom row (G-I) in the presence of peptide 2 (SKRRVPNGPDPIHN). Plant growth is shown on day 6 after treatment (left column; A, D, G), on day 20 after treatment (middle column; B, E, H) and on day 20 of recovery, whereby seedlings were treated for 6 days and then transferred to plates without peptide for the remaining 14 days (right column; C, F, I). Bar on the bottom of each column indicates 2 cm.

**Figure 5:**
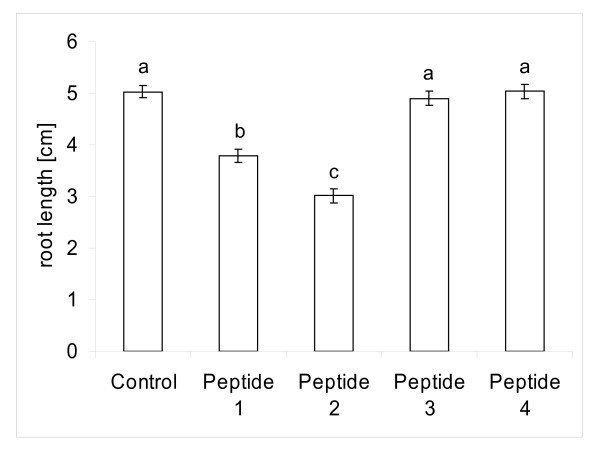
**Sequence specificity of CLE peptide activity**. Root length of *Medicago truncatula *plants at 6 days after treatment with different peptides. Control plates did not contain peptide, peptide 1 (SKRKVPSCPDPLHN) and peptide 2 (SKRRVPNGPDPIHN) resemble the CLE motif, peptide 3 (randomized version of peptide 1, DHKSKPPVLRPNSC) and peptide 4 (randomized version of peptide 2, PVHPKGNRNDISPR) do not resemble the CLE motif. Bars with different letters differ significantly at p < 0.0001 (N = 27; one-way ANOVA). Both CLE peptides are significantly different from the no-peptide control and the control peptides with randomized amino acid sequence.

*M. truncatula *seedlings were grown with the peptide as growth media additive [22]. A termination of root growth was clearly observable six days after treatment in all of the seedlings treated with peptides 1 and 2 compared to control plants in the absence of either peptide and compared to the randomized peptides (Figure 4, Figure 5). After six days of treatment, root growth of the plants treated with peptide 1 and peptide 2 was significantly (p < 0.0001, one-way analysis of variance) reduced compared to the no-peptide and the random peptide controls. After 20 days, almost no further root growth was observed in seedlings treated with peptide 1 or 2. We noted an increased formation of lateral roots in both peptide treatments. Similar to the RAM, the newly formed meristems of the lateral roots terminated their growth shortly after lateral root emergence. We tested the reversibility of the peptide treatment by transferring half of the plants to a fresh plate not containing peptides. The RAM recovered within two weeks. In some cases the main root terminated its growth, and a lateral root elongated instead. We also observed that the main root could recover its growth after release from the peptide-containing medium. In this experiment, shoot growth was not noticeably affected by the presence of peptide in the agar, although shoots were not in direct contact with the agar.

## Discussion

### Identification of CLE members

The aim of this study was to identify new members of the CLE signaling peptide family in plants, in particular from legumes. The overall criteria for assignment of candidates to the family were stringent and limiting, allowing us to eliminate many false positive hits. The number of redundant sequences retrieved from our custom database was much larger than the number of sequences in the final non-redundant set. This indicates that, in many cases, several redundant sequence entries from EST and genome databases were combined under one CLE number. That the same CLE sequences were reproducibly recovered from both EST and genomic data makes it highly likely that these proteins are actually expressed in the plant. However, the number of CLE signaling peptides identified from plant species with a sequenced genome so far cannot be considered complete. This is because our analysis was based on the proteins predicted from the genome, which are annotated by automated open reading frame detection. This automatic detection frequently fails to detect small proteins like members of the CLE family [38-42]. As such we would expect improvements in prediction of expressed proteins to, possibly, identify further CLE signaling peptides. The set of sequences that we were able to identify consisted of 65 known and 114 new CLE sequences bringing the number of identified potential CLE signaling peptides to 179. The dataset included 28 new legume CLE sequences. Sequence similarity of the CLE family was analyzed not based on phylogenetic trees but on pairwise sequence comparisons. As pointed out by Floyd and Bowman, the restricted sequence conservation of 14 amino acids hampers phylogenetic analysis in case of the CLE family [56].

So far, we were able to identify one representative of the CLE family from *Physcomitrella patens *using the EST database, although more might be found once the genome of this organism is made publicly available. From the green alga *Chlamydomonas reinhardtii*, of which we used the genome as well as the EST database and TIGR transcript assemblies, we could only identify one CLE sequence, which did not cluster with any of the groups (Figure 2). The biological function of this putative CLE signaling peptide in *Chlamydomonas *will need to be established in future studies. It will be interesting to find out if the CLE sequence of *Chlamydomonas *has a different role to the function of CLE signaling peptides in higher plants, which show cell differentiation and meristem activity, and whether CLE signaling peptides are part of an essential genetic equipment required for plant development [56].

A new finding was the identification of CLE protein sequences carrying multiple CLE motifs. We were able to detect multidomain CLE proteins carrying two to six motifs from *O. sativa*, *T. aestivum *and *M. truncatula*, but not in any other plant species. The sequences originated from different databases and sequencing projects. To reduce the probability that mis-assembly of the genome or TC-entries is responsible for the occurance of proteins containing multiple CLE-domains, we examined the genomic positions and EST coverage of the proteins. Using the TIGR *O. sativa *genome browser, we determined that the motifs in CLE75 and CLE76 originated from a single exon. Examining the TC-entries for CLE30 and CLE32 from *T. aestivum *we were able to find 25 individual sequence reads (EST's) for CLE30 and five sequence reads for CLE31 covering at least two CLE motifs. This provides evidence that both of the multi-CLE proteins from *T. aestivum *are transcribed in the predicted manner and are unlikely to be an artifact of TC-assembly. We hypothesize that the full protein sequence releases several active signaling peptides after processing, which could provide an amplification effect.

### Clustering of CLE motifs and identification of new secondary motifs

Cluster analysis of the CLE sequences using CLANS showed that these sequences could be assigned to 13 groups. The grouping we observed based on sequence similarity corresponds to the classification of ectopic CLE overexpression phenotypes in *A. thaliana *made by Strabala *et al.*[12]. Furthermore, it is equivalent to the phylogenetic grouping and consistent with observations of effects on the root apical meristem and tissue differentiation [8,24]. We observed a close spatial arrangement of known functional orthologs in the graph (e.g., FON4 and CLV3, see group 3, Figure 2) [26]. The established grouping allows the interspecies identification of further orthologs. We hypothesize that CLE125, located in the same group as CLV3 and FON4, is the functional ortholog of CLV3 in *P. trichocarpa*, and CLE143 and/or CLE147 in *Z. mays*, respectively. The grouping also allows narrowing down the number of candidate CLE genes from which the nematode *H. glycines *may have acquired its CLE signaling peptide. The *H. glycines *CLE sequence clustered tightly with group 2. Provided a lateral gene transfer occurred, this points to the nematode having acquired a CLE member from group 2 and may allow insights as to the functions of group-2 CLE signaling peptides as well as to the function of the *H. glycines *CLE signaling peptides. Overall, the results indicate that there is a connection between the sequence similarities leading to distinct groups of CLE members and the observed effect in case of excess peptide supply (ectopic expression or peptide addition) [8,12,21-24]. However, as ectopic expression might lead to phenotypes that do not reflect the *in vivo *role of CLE signaling peptides, future studies could focus on characterizing the exact biological function of each signaling peptide.

In a peptide assay we confirmed that two *in silico *identified signaling peptides had biological function in *M. truncatula*. Both peptides arrested the activity of the root apical meristem and lateral root meristems, resulting in reduced root growth. The sequences of these peptides were found in CLE members grouping either in group 7, 8 or 9 (Figure 1). Other CLE peptides that clustered in these groups were also found to have a negative effect on the root apical meristem, for example CLE25 and CLE26 in studies in *A. thaliana *and *Zinnia elegans *[8,24]. In addition, members of CLE sequences in group 9, including CLE9–CLE13 also showed an effect on the RAM [8,24].

One of the main questions remaining is why plants encode such a large number of LRR-RLKs, and what their function and ligands are. CLE signaling peptides could bind to LRR-RLKs related to the CLV1/CLV2 receptor, but so far little is known about specificity between CLE peptide ligands and their receptors. Group specific and invariant residues as well as variations of conserved residues identified through sequence analysis could determine a selective specificity for receptor subgroups targeted by a given signaling peptide. Furthermore, our cluster analysis revealed that there were regions outside the CLE motif that correlated in sequence similarity with the groupings generated by CLANS based on the primary CLE motif sequence. It has been shown that processing occurs in members of the family, meaning that one or a complex of enzymes recognize part of the protein sequence and cleave it. The addition of a single arginine residue at the C-terminus of the conserved domain results in a decrease of peptide activity [8,24]. This shows that correct cleavage and specific recognition of the conserved domain are required for the maximum activity of the signaling peptide. The process and detailed mechanism remain unknown. Furthermore, it is unclear whether all peptides are processed and modified in a manner equivalent to CLV3 and TDIF, which were found to be active as 12 amino acid peptides. We hypothesize that the extensions of the motif may be involved in the specific recognition and processing of the signaling peptide precursor.

## Conclusion

We identified 114 new CLE domain proteins from a variety of plant species, including 28 new sequences from legumes, which could be potential ligands for the LRR-RLKs controlling nodulation. We also found several CLE proteins with multiple CLE domains, which could represent a mechanism for peptide signal amplification. Clustering of the sequences showed 13 distinct groups, which were found to have conserved secondary motifs outside the CLE domain. Biological activity of two of the predicted signaling peptides were confirmed *in vivo*. CLE signaling peptides could have potential biotechnological applications for altering plant development, as exemplified in US patent No. 7179963 using CLE signalling peptide functions in *Z. mays*. While we could not test the biological activity of all the identified signaling peptides in our study, we hope that the CLE domain proteins presented in this study will allow other researchers to test their function in a variety of plant species and as potential ligands of LRR-RLKs.

## Methods

### Biological sequence resources

Several sequence resources were combined, forming a custom, redundant protein database. Expressed Sequence Tags (EST) databases from *A. thaliana *(release 12.1), *Brassica napus *(release 1), *C. reinhardtii *(release 5), *G. max *(release 10), *Lotus japonicus *(release 3), *Lycopersicum esculentum *(release 10.1), *M. truncatula *(release 8), *Nicotiana tabacum *(release 2), *O. sativa *(release 16), *Solanum tuberosum *(release 10), and *Z. mays *(release 16) were downloaded from the TIGR Gene Indices (now available at the Dana-Farber Cancer Institute gene index project) [49]. TIGR Transcript Assemblies (TA) from *A. thaliana*, *Brassica napus*, *C. reinhardtii*, *P. patens*, *G. max*, *Glycine soja*, *Lotus corniculatus*, *Lupinus albus*, *Lycopersicum esculentum*, *M. sativa*, *M. truncatula*, *Nicotiana tabacum*, *O. sativa*, *Phaseolus coccineus*, *Phaseolus vulgaris*, *Pisum sativum*, *Solanum tuberosum*, and *Z. mays *were added to this set (all release 1, 15 August 2005) [50]. The proteins predicted from the plant genomes of *A. thaliana *(NCBI Genbank release 5, 03 May 2006) [57], *C. reinhardtii *(JGI, release 3) [58], *M. truncatula *(Genome Sequencing Project release 17 July 2006) [59], *O. sativa *(release 4, 30 December 2005) [60], and *P. trichocarpa *(JGI, release 1) [61] were also included.

Sequence names were truncated to a unique identifier. Information about the database origin of each sequence was added to the unique identifier (i.e. OS-TA, OSEST, OSGEN for *O. sativa *TA, EST or genomic sequences respectively). Nucleotide sequences were translated into protein sequences in all six reading frames (universal code), and frame information was appended to the sequence identifier (e.g. "_+2"). The translated nucleotide sequences and modified protein sequences derived from genomic data were combined into a single file and formatted using Formatdb (options: -p T and -o T) [43]. The resulting database contained 3,631,558 sequences. To determine whether CLE sequences were specific to plants, a separate search was based on the non-redundant protein database (NCBI nr, version 15 June 2006.).

### Query sequences

A set of 45 known CLE sequences (CLE1 – CLE17, CLE19 – CLE44, HgCLE, and CLV3; retrieved from Genbank and TIGR) were combined in a FASTA-file, aligned using CLUSTALW 1.83 and manually refined [51]. From the multiple sequence alignment, a profile hidden Markov model (HMM) was build using HMMer 2.3.2 [47]. The original FASTA-file was re-aligned to the HMM (HMMalign) and verified using the alignment editor AlnEdit [62] to check for consistency of this realignment step. The alignment revealed a region of high conservation of 12–18 amino acids at the C-terminus that consistently matched the HMM (corresponding to "HEELRTVPSGPDPLHH" of CLV3). We therefore decided to extend the "conserved domain" beyond the 12 amino acids defined previously [5,24,25]. This alignment (iteration 0) served as input for iteration 1 to HMMaccel. Additionally, the 12–18 amino acid stretch that matched in the alignment was extracted and used to build an HMM consisting solely of the conserved region. HMMaccel is available for download [48].

### Motif search of the plant database

Each iteration started with a plain FASTA-file (the output of the previous iteration). All sequences in the FASTA-file were aligned against the HMM of the conserved domain. The resulting alignment was verified using AlnEdit and converted into aligned FASTA-format (for input to HMMaccel). Full-length sequences were retrieved for all HMMaccel hits and re-aligned to the HMM of the conserved domain. The resulting alignment was manually examined (AlnEdit) and converted to aligned FASTA-format (input to HMMaccel for the next iteration).

The settings for PSI-BLAST throughout iteration 1 and 2 were a cut-off E-value of 10,000 (parameter -e), an E-value threshold of 0.005 for the inclusion of sequences (parameter -h), 250 for the numbers of displayed high scoring sequence pairs (parameter -b) and 500 for the numbers of displayed hits (parameter -v). The parameters -b and -v were altered in iteration 3 and iteration 4 to -b 1 and -v 1,000. The parameters for HMMer in HMMaccel caused hits up to E-values of 10 to be returned and the HMMs to be calibrated using 5000 samples.

We observed a large number of false positive sequences that were added to the dataset after iteration 3 compared to previous iterations. Without removal of these sequences, the dataset became inaccurate in iteration 4. To avoid a biased removal of sequences and for a reproducible optimization of the sequence set, cluster analysis of sequences (CLANS) was used [52,53]. The conserved region of the 811 hits of iteration 3 was extracted and analyzed in CLANS. A total of 312 sequences were discarded as false-positives from the sequence set. The remaining 499 sequences were submitted to a final iteration. The HMM derived from these 499 sequences is available as Additional File 3. After iteration 4 the dataset consisted of 659 protein sequences. The large number of false positive hits returned in iteration 3 point to the method having reached the limits of what it could resolve. After removal of false positives, a fourth iteration was performed to reduce the number of false negatives. The aim of the iterative search was to find all CLE peptides in the database and therefore false negatives were of greater concern than false positives.

The species and database origin was contained in the sequence identifier. The full annotation information of the sequences was subsequently retrieved from the original FASTA-files. The calculation of isoelectric points and molecular weights was performed with PROT STATS from the EMBOSS 4.0.0. package [63]. Protein length, position and sequence of the CLE domain were extracted from the FASTA-file.

### Sequence analysis

Full-length protein sequences as well as conserved domains were analyzed in CLANS [52]. The dataset was spiked with the 45 original query sequences to check their positioning and group assignment in CLANS. Using the full-length sequences, we found several sequences with more than one domain, which we noticed by their behaviour in CLANS. Grouping of CLE peptides was observed in the cluster analysis of the conserved domain. The individual groups were extracted and aligned using Kalign [64]. The alignments of primary CLE motifs, their extension and additional motifs were visualized with WebLogo 3.0b14 to represent all sequences of the group [65].

### Peptide synthesis

Peptide 1 (SKRKVPSCPDPLHN) and peptide 3 (randomized peptide 1, DHKSKPPVLRPNSC) as well as peptide 2 (SKRRVPNGPDPIHN) and peptide 4 (randomized peptide 2, PVHPKGNRNDISPR) were synthesized with >75% purity by GL Biochem (Shanghai, China). The peptides carried a free carboxyl acid group at the C-terminus. Peptide 1 and 2 were designed according to the CLE motif, peptides 3 and 4 do not resemble the CLE motif as the sequences are randomized versions of the amino acid sequence of peptide 1 and peptide 2. Randomized sequences were generated with the RandSeq tool at ExPASy [66]. Peptides were diluted to a final concentration of 10 μmol/l [22] in sterile, nitrogen-free Fåhraeus media [67].

### Peptide assay

Wildtype *M. truncatula *cv. Jemalong A17 seeds were scarified on fine sand paper and sterilized using 80% technical grade ethanol (5 min), 6.25% sodium hypochlorite solution (5 min), and freshly prepared 200 mg/l Augmentin^® ^Duo (Amoxicillin/Potassium Clavulanate; GlaxoSmithKline, Brentford UK) (5 h) with five washes of sterile Milli-Q^® ^water (Millipore, Billerica USA) between treatments. Seeds were germinated on Fåhraeus agar plates without the presence of peptides at 4°C (12 h) and 28°C (24 h) in the dark [68]. Seedlings were briefly washed with sterile, phosphate-buffered saline before transfer to a fresh plate (10 seedlings per plate) containing peptide or no peptide (control). Plates were sealed with Parafilm M^® ^(Structure Probe Inc., West Chester USA) on the bottom half and grown in an upright position. Black paper carton was placed to cover the bottom 2/3 of plate to minimize light exposure to roots. Plants were grown at constant 25°C and 100 μE light intensity under extended day conditions (16 h day/8 h night) [68]. Root growth was measured every 24 h for six days, starting on the day of transfer (t = 0d). To test the reversibility of the peptide treatment (t = 6d), half of the plants (five) were transferred from the plate containing the peptide to a fresh media plate (without peptide) and grown for two weeks (t = 20d). Photographs were taken at time points 6 d and 20 d. Statistical analyses were performed using GenStat^® ^9.2 (VSN International Ltd, Hemel Hempstead UK).

## Authors' contributions

KO carried out the bioinformatic analysis and peptide assays. NG designed the database and contributed with programming. GFW and PMG were involved in the overall design and coordination of the experiments. UM performed statistical analysis and some of the peptide assays, TF conceived the strategy of the motif search and contributed to the programming and the overall experimental design. All authors read and approved the final manuscript.

## Supplementary Material

Additional file 1 Table of Identification. Click here for file

Additional file 2 Multiple sequence alignments of groups and full length sequence logos. Click here for file

Additional file 3 **last HMM**. Hidden Markov model after the third iteration, generated by HMMer 2.3.2. Click here for file
