# Bioinformatic analysis of the CLE signaling peptide family

**DOI:** 10.1186/1471-2229-9-17

**Published:** 2009-02-09

**Authors:** Karsten Oelkers, Nicolas Goffard, Georg F Weiller, Peter M Gresshoff, Ulrike Mathesius, Tancred Frickey

**Affiliations:** 1 School of Biochemistry and Molecular Biology, The Australian National University, Canberra, ACT, Australia; 2 Research School of Biological Sciences, The Australian National University, Canberra, ACT, Australia; 3 The University of Queensland, Brisbane, QLD, Australia; 4 The Australian Research Council Centre of Excellence for Integrative Legume Research, The University of Queensland, Brisbane, QLD, Australia

## Correction

After the publication of this work [1] we became aware of the fact that in Supplementary File 2 some of the sequence identifiers (i.e. CLE numbers) of the *Medicago truncatula *CLE protein sequences in the multiple alignments were incorrect. Table 1 shows the corrected CLE numbers. Additional file 1 and Additional file 2 show a full corrected list of all CLE peptides with their corresponding groupings. The tables within these files also include identifiers for those sequences that were not grouped in Figure 2 of the original article [1].

**Table 1:** Corrected CLE numbers for *Medicago truncatula *CLE peptides

Suppl.2	Corrected	Corrected	Suppl.2
CLE35	CLE71	CLE35	CLE69
CLE36	CLE74	CLE36	CLE66
CLE37	CLE39	CLE37	CLE74
CLE38	CLE65	CLE38	CLE71
CLE39	CLE70	CLE39	CLE37
CLE64	CLE67	CLE64	CLE65
CLE65	CLE64	CLE65	CLE38
CLE66	CLE36	CLE66	CLE72
CLE67	CLE69	CLE67	CLE64
CLE69	CLE35	CLE69	CLE67
CLE70	CLE73	CLE70	CLE39
CLE71	CLE38	CLE71	CLE35
CLE72	CLE66	CLE72	CLE73
CLE73	CLE72	CLE73	CLE70
CLE74	CLE37	CLE74	CLE36

The table shows the CLE numbers in the original Supplement 2, and the corresponding corrected numbers.

We regret any inconvenience that this inaccuracy in the data might have caused.

## Supplementary Material

Additional file 1 **Full listing of CLE peptide numbers for each group of CLE peptides, sorted by CLE peptide number.** Table: The groups correspond to those shown in Figures 2 and 3 of the original article [1]. Click here for file

Additional file 2 **Full listing of CLE peptide numbers for each group of CLE peptides, sorted by CLE peptide groups.** Table: The groups correspond to those shown in Figures 2 and 3 of the original article [1]. Click here for file
