# Supplementary material for: Bioinformatic analysis of the CLE signaling peptide family
Source: BMC Plant Biol. 2008 Jan 3;8:1. doi: 10.1186/1471-2229-8-1 (PMC2254619; doi:10.1186/1471-2229-8-1)

Group1
3 members

```
LADDER                         10|          20|          30|          40|          50|          60|          70|          80|          90|          100|          110|          120|          130|
CLE106_Oryza sa    --------------------------------MAIRSCVCIVLC---LVLVVVVGLAATQAEAARALAEYAAPPG---GDYDDDVAGGGGGGFGIRGRRRPG-RWNV-RSLQGG---KREVPGGPDPQHHY
CLE108_Oryza sa    --------------------------------MDGQDRSNGKLCRRPQRQRMRTQELAAAPEARRIV--YAAAVGYSTGACEGGVAGNGGGGA--------QPRFKWNTRREIGGD---KRTVPGGPDPQHHH
CLE152_Zea mays    GHDTERRNTPALGSRSPQVLRWLQTFSLRSTPMAARVGI-VVLC--SLLLLLIVG--AATAAEARVV-----PPGYAAPDIDVARAAGTGGGR---RGGIPRGSRWNV-QSVQGDSARKREVPGGPDPQHHG
LADDER                         10|          20|          30|          40|          50|          60|          70|          80|          90|          100|          110|          120|          130|
```

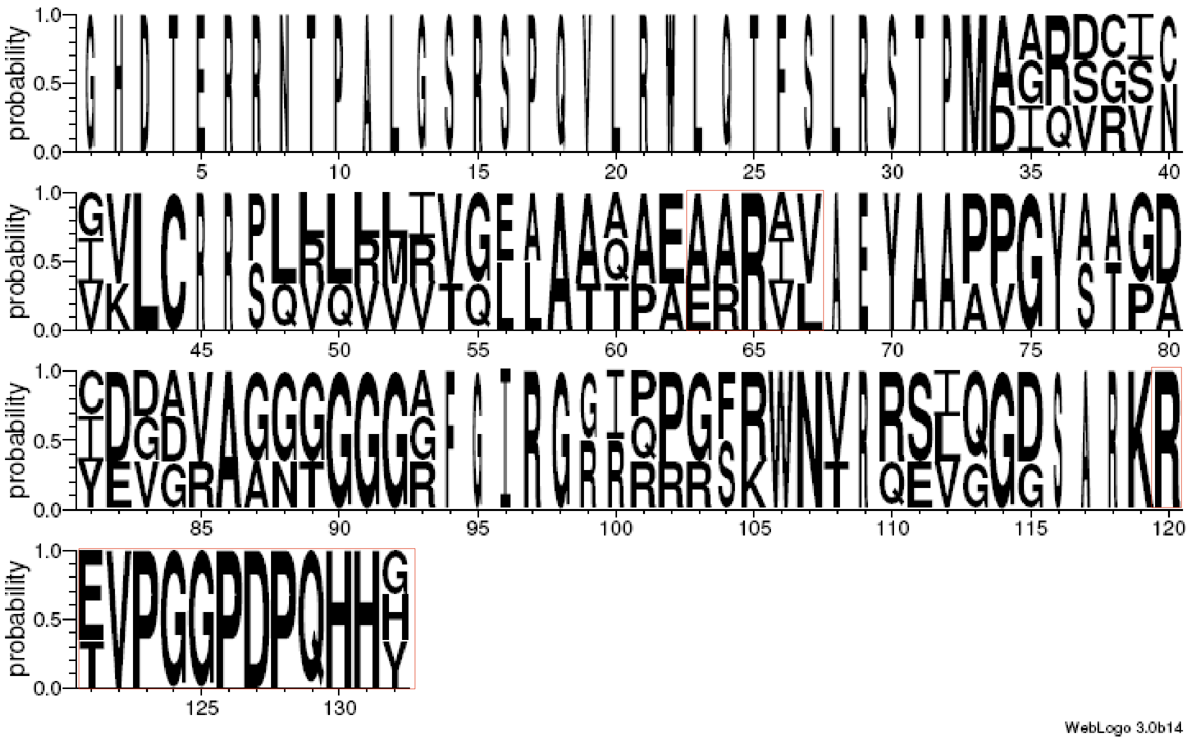

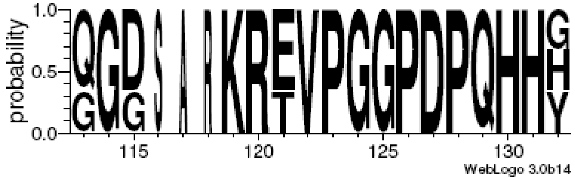

Group2
18 members
dwarf 21
HgCLE

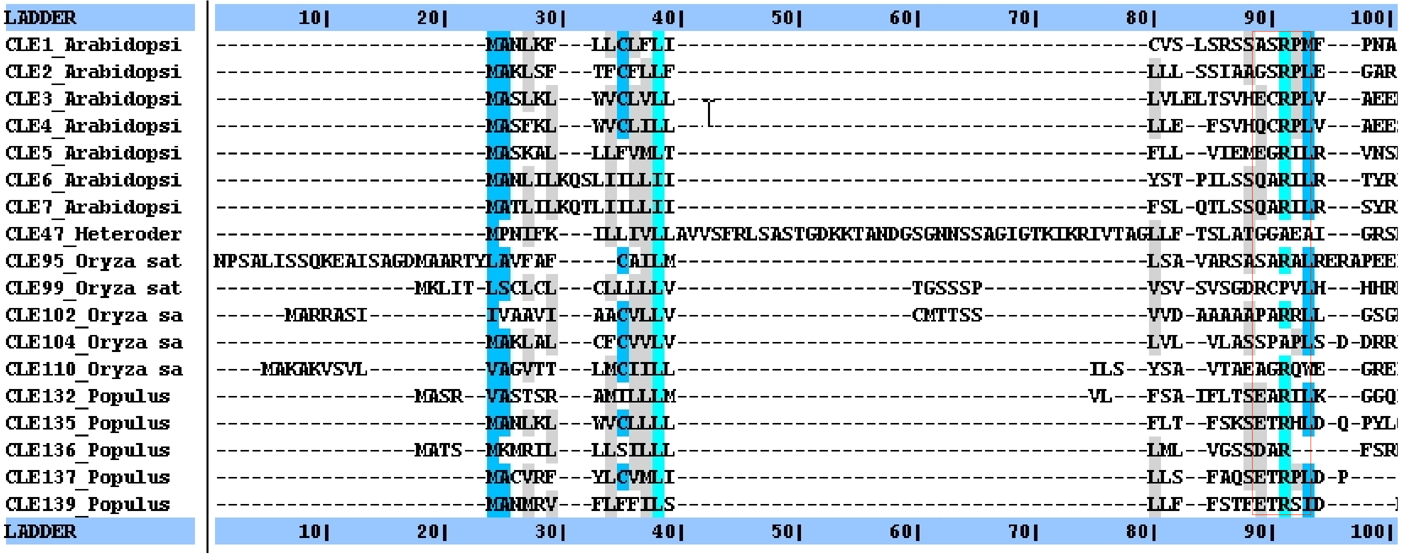

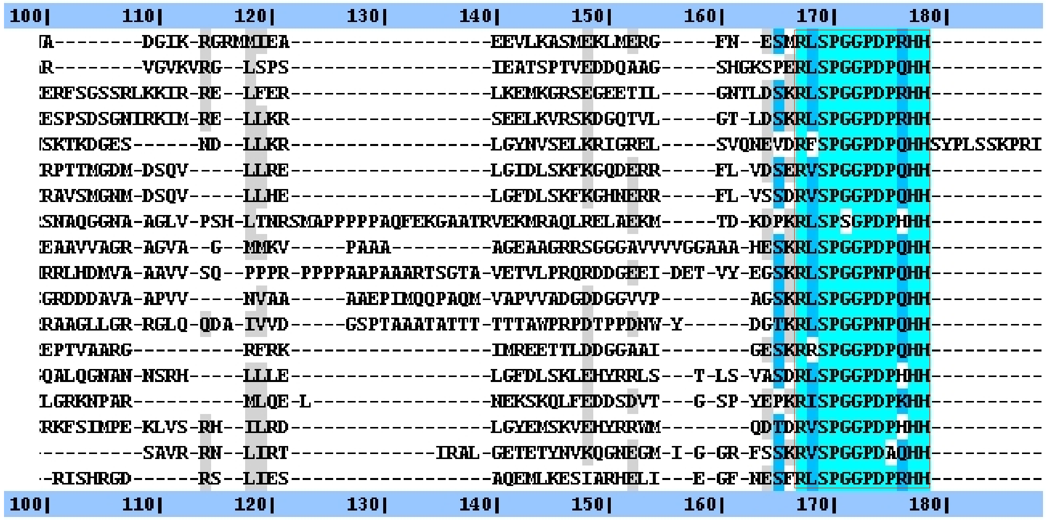

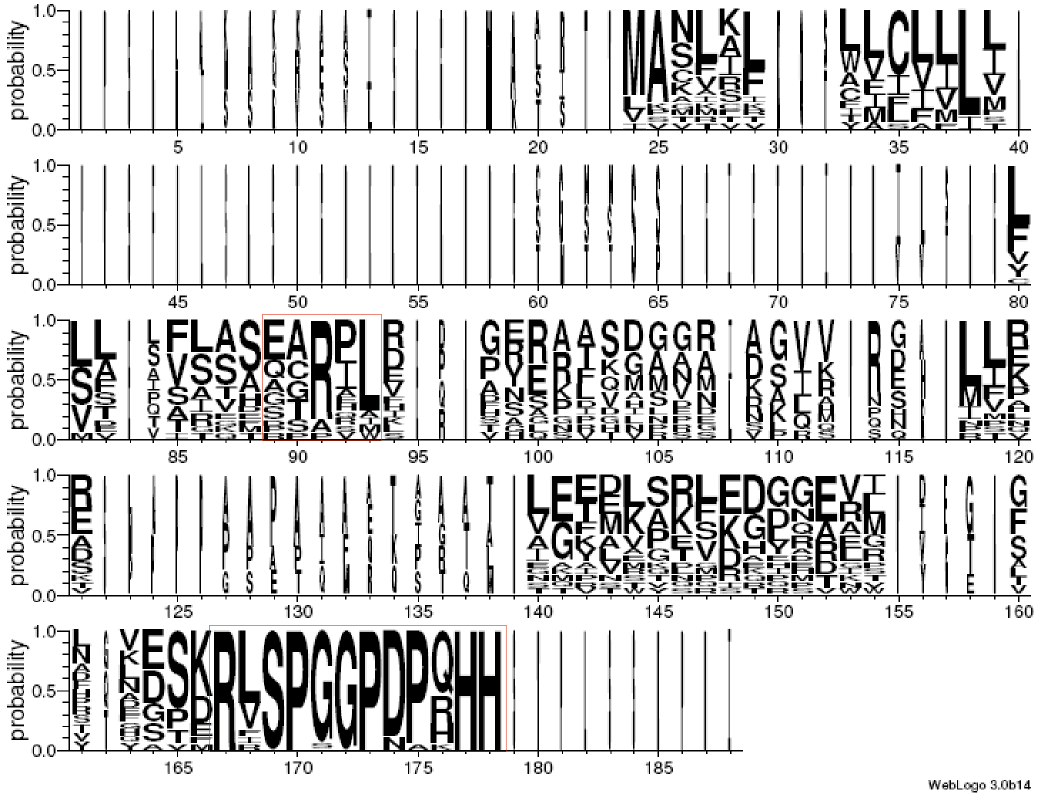

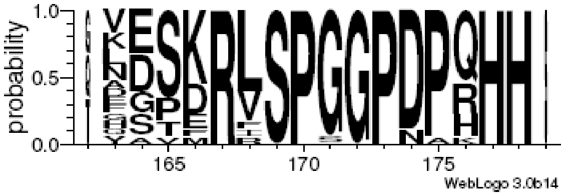

Group3
5 members
CLV3, fon4

```
LADDER                        10|       20|       30|       40|       50|       60|       70|       80|       90|      100|      110|      120|      130|      140|      150|
CLE46_Arabidops   MDS--KSFLLLLLLFCFLFL------HDASDLTQAHAHVQGLSNRKMMMMKME-----------------SEWVGANGEAEKAKTKGL---GLHEELRTVPSGPDPLHHHVNPPRQPRN--NFQLP-------------------------------
CLE48_Oryza sat   M-G--RLFLCLVVAWCWVALLLVAPVHGRVGLPGEFSGDQRPVPATSFDLVTEPKTKQPRGVKGTRRPSWSSWSSTASRSSPPPGRGAPSAAAAAAELRSVPAGPDPMHHH-GSPRRPEHARSTGRP-------------------------------
CLE125_Populus    M-ACPSKFYSLMLVLVLFYMVMEE--SYGLNLSQSLSLHGCSTGQRCFYAEAV----SPVDVKSRK----VLVVLTGGLRGPTGSTGNGEKLEIRELRAAPSGPDPLHHNGGSPEKPRT------P-------------------------------
CLE143_Zea mays   --------------------------------STSVNANVWQTDEDAFYSTNK------LGVNGNMEMA-QQQGGFIGHRPRLASFNRASKQLDREKRPVPSGPDPIHHS-IPSHAPQH------PPSYGKAPYEDDKSIASPGLSNLIGPPPFLDR-
CLE147_Zea mays   -----------------------------------------------TRTDDK------PGVNRNMEMQ-QQQGGFIGHRPRLASFNRASKQLDREKRPVPSGPDPIHHS-IPSHAPQH------PPSYGKAPYEDDKSIASPGLSNLIGPPPFLDRY
LADDER                        10|       20|       30|       40|       50|       60|       70|       80|       90|      100|      110|      120|      130|      140|      150|
```

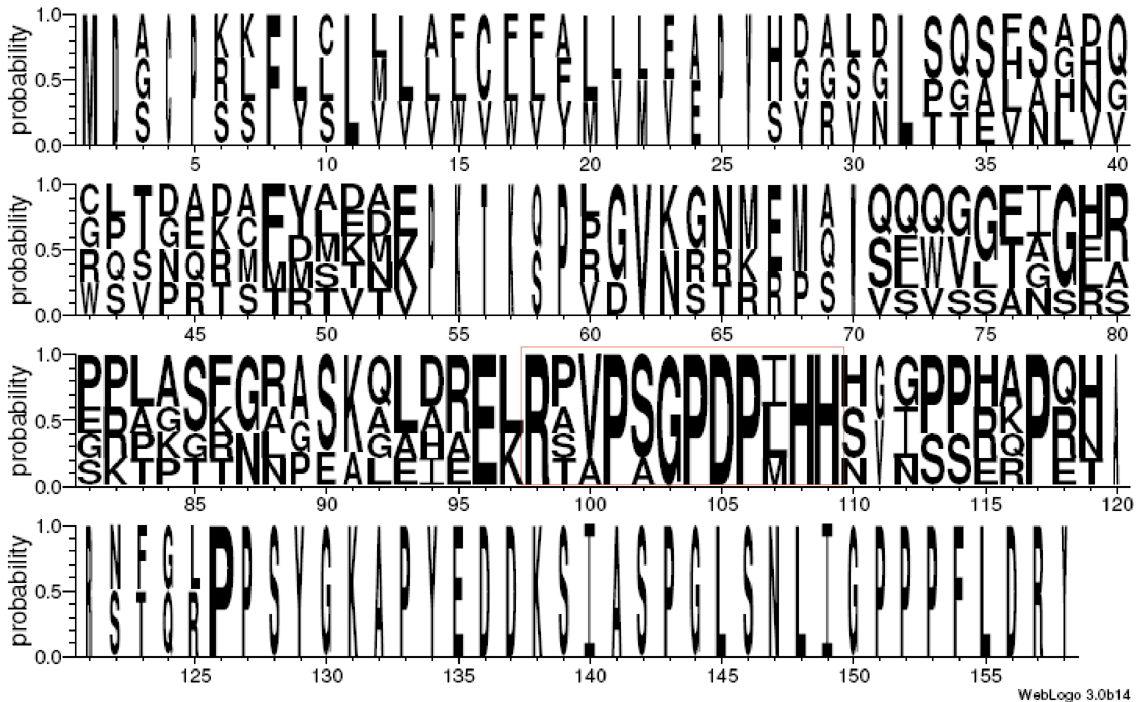

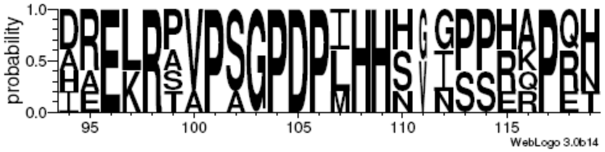

Group4
5 members

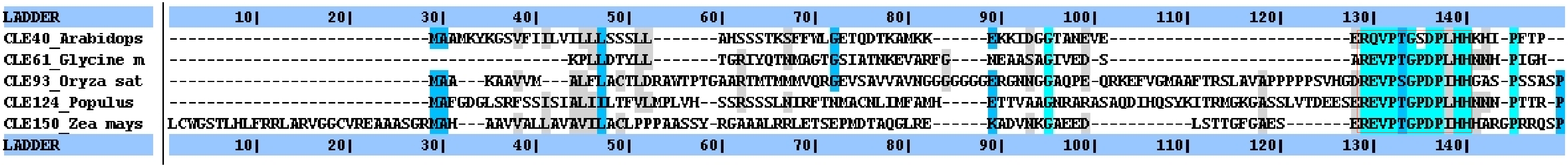

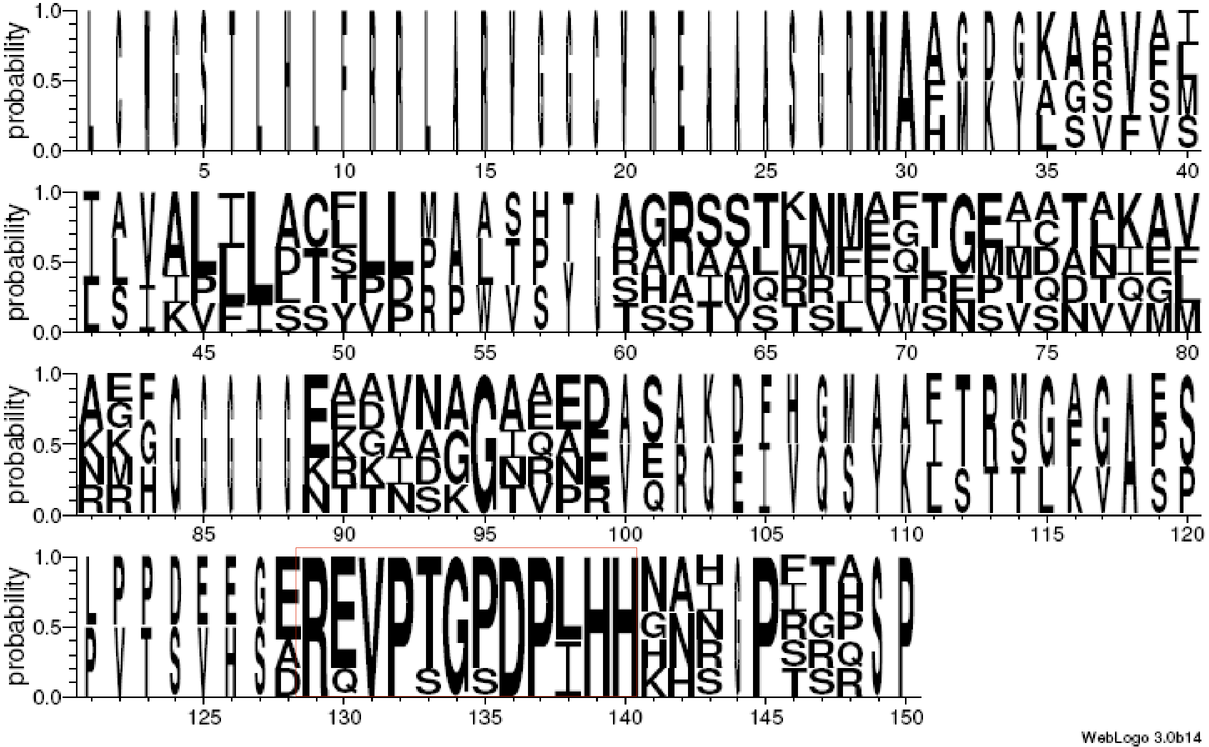

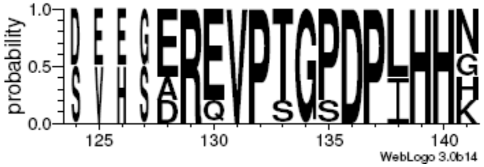

Group5
18 members
shrub-like
TDIF

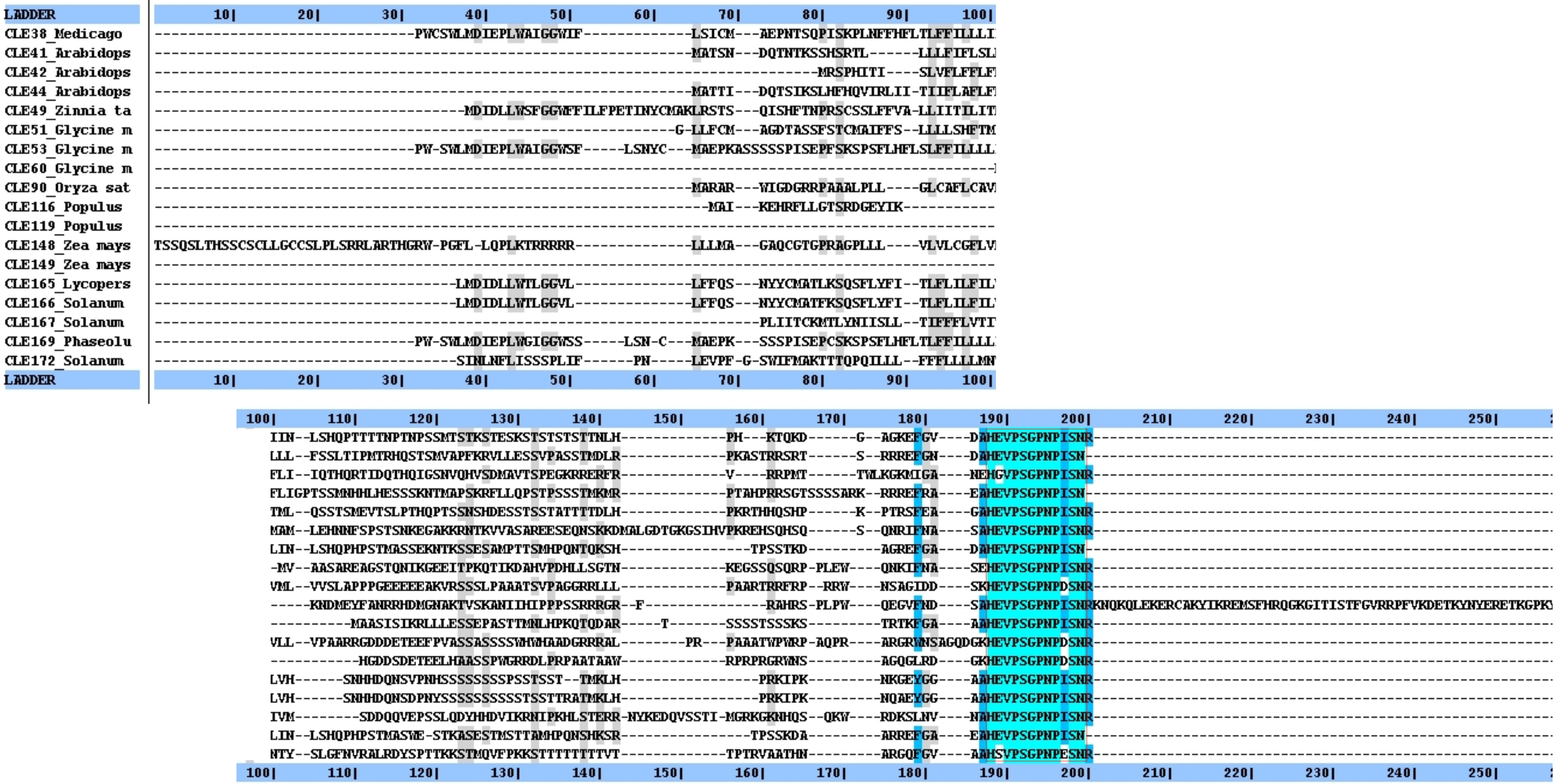

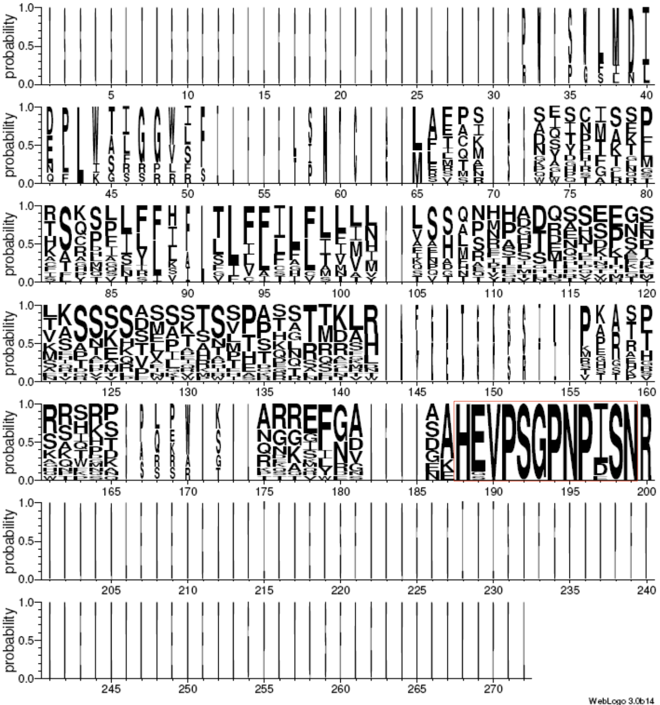

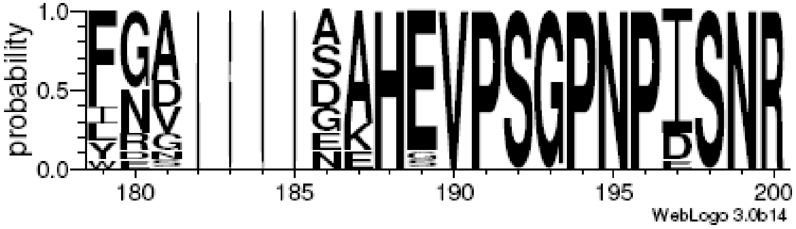

Group6
4 members

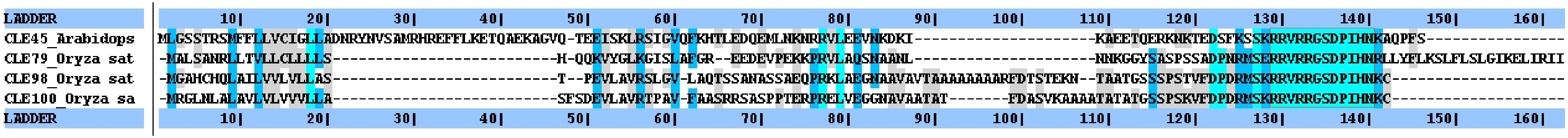

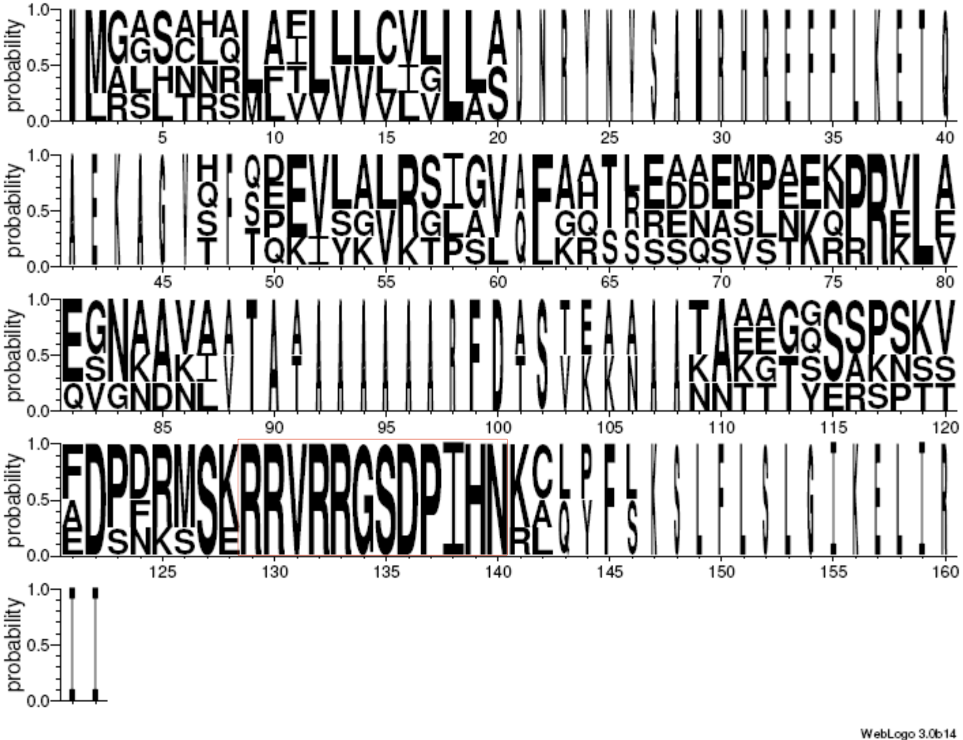

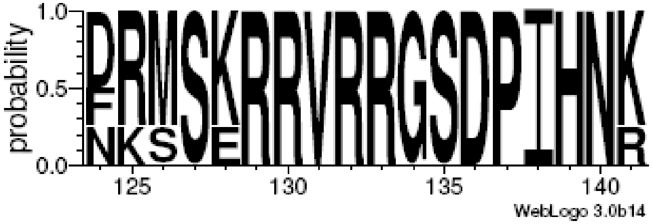

Group7
14 members
long root

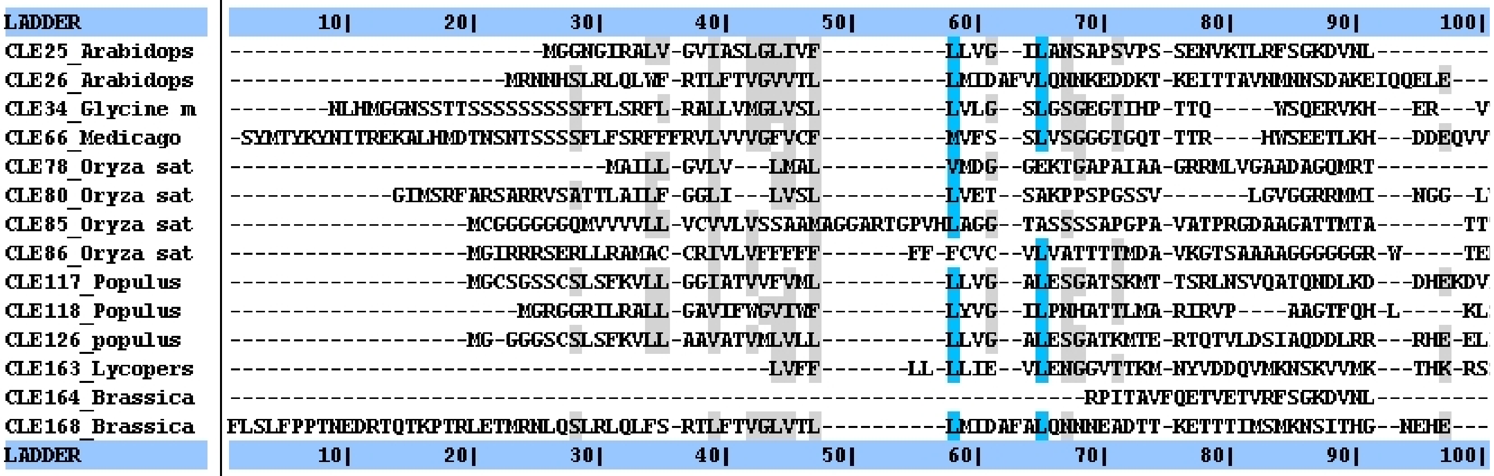

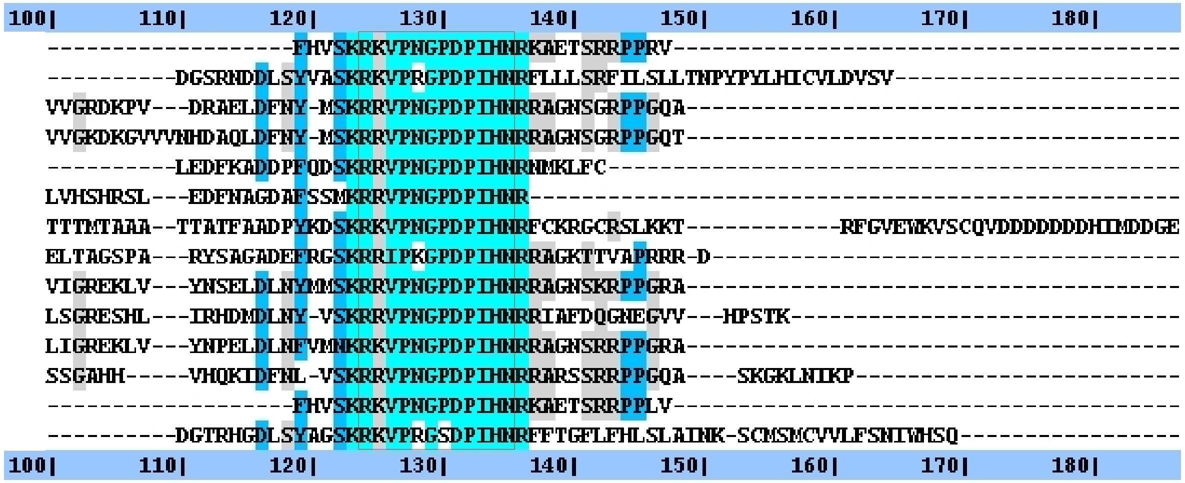

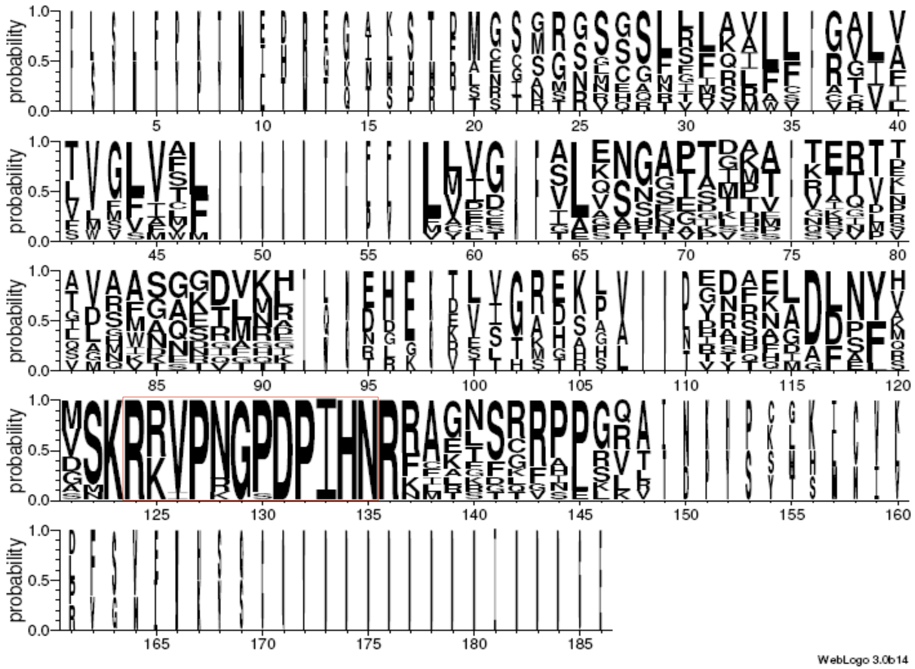

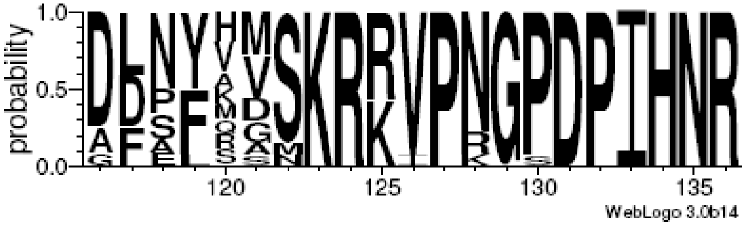

Group8
6 members

```
LADDER                 10|       20|       30|       40|       50|       60|       70|       80|       90|      100|      110|      120|
CLE24_Oryza sat    ---------------------MPPPPATTPLPRRRLALILCLAWALWLH-GGGGGISLADAFQAPTPARLSSGSSYAVG-----SRPVPAAAPRWSSSSASEAAARF-------ADDKRRIPSCPDALHNR
CLE27_Arabidops    ---------------------MTHAREWRSSLTTTLLMVILLSYMLHLF-CVYSRVGAIRIFPE-TPAS---------G-----KRQEEDLMKKYFGAGKFPPVDSF--VGKGISESKRIVPSCPDPLHN-
CLE55_Glycine m    YLVPRKRVFGNWVQERPKIYVFYSLAMLVAGGKRLLYVSVVVMLVVLHMCHTCQVGAIRVFPGNAVAN----VEFSHGNMDNNKSKEEDLFKKYFSGRTHLGPSNT--TQKGFDDSKRRVPSCPDPLHN-
CLE64_Medicago     ----------------------------------------------------------------------------ARG---QDVMHNKDLLNKYFKGRTFFGSSNKNETQMGFDDSKRKVPSCPDPLHN-
CLE77_Oryza sat    -MRPARRGWGV----------GALARAFLVL----LLLLLAAATTTTIG-FGCRGAEAIRVIPPHGPAPGSARSSRGHG-HRRSHGNAARVVDAAMPVVGTRPVPAL--SPAADEESKRRIPSCPDPLHNR
CLE176_Phaseolu    -------------------------------------------------------VQGIRVLPRNAQPN----VEFSHGNINDDNNNNDDLFNKYFNGRTPLAPTNT--IQKGFDETKRILPSCPHPLHN-
LADDER                 10|       20|       30|       40|       50|       60|       70|       80|       90|      100|      110|      120|
```

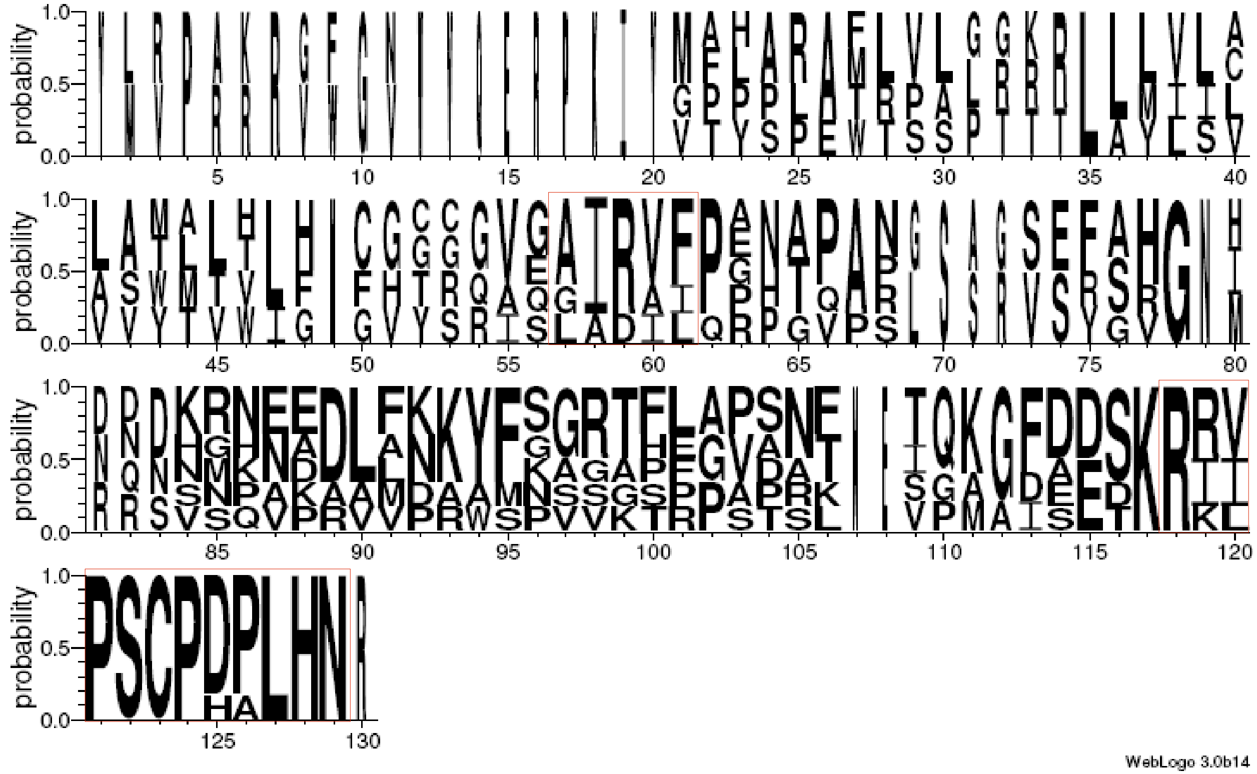

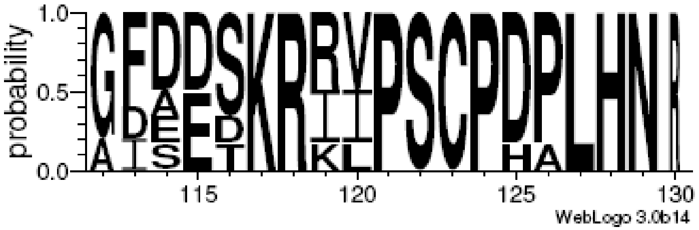

Group9
17 members
wus-like

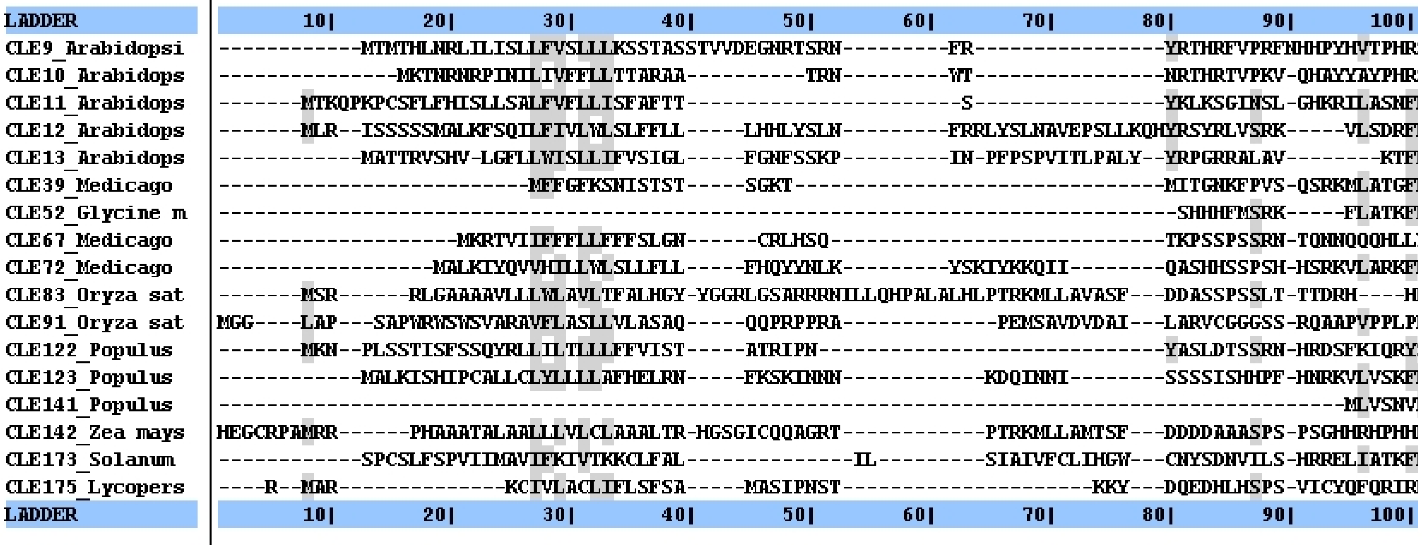

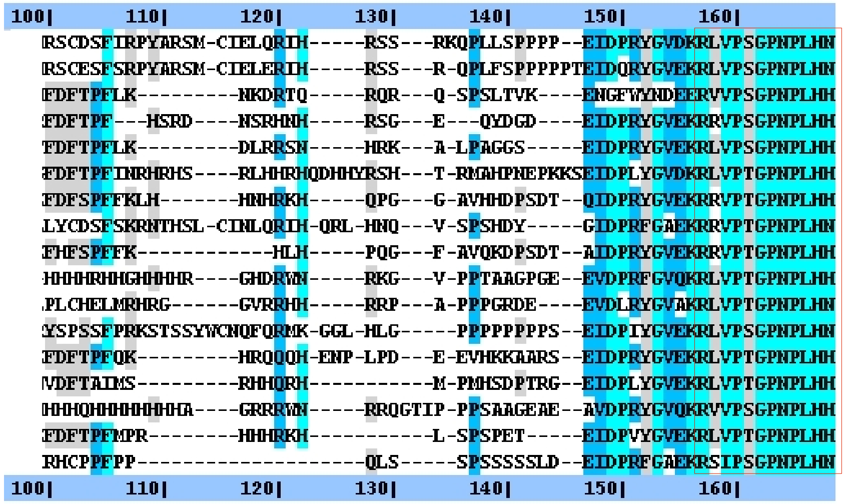

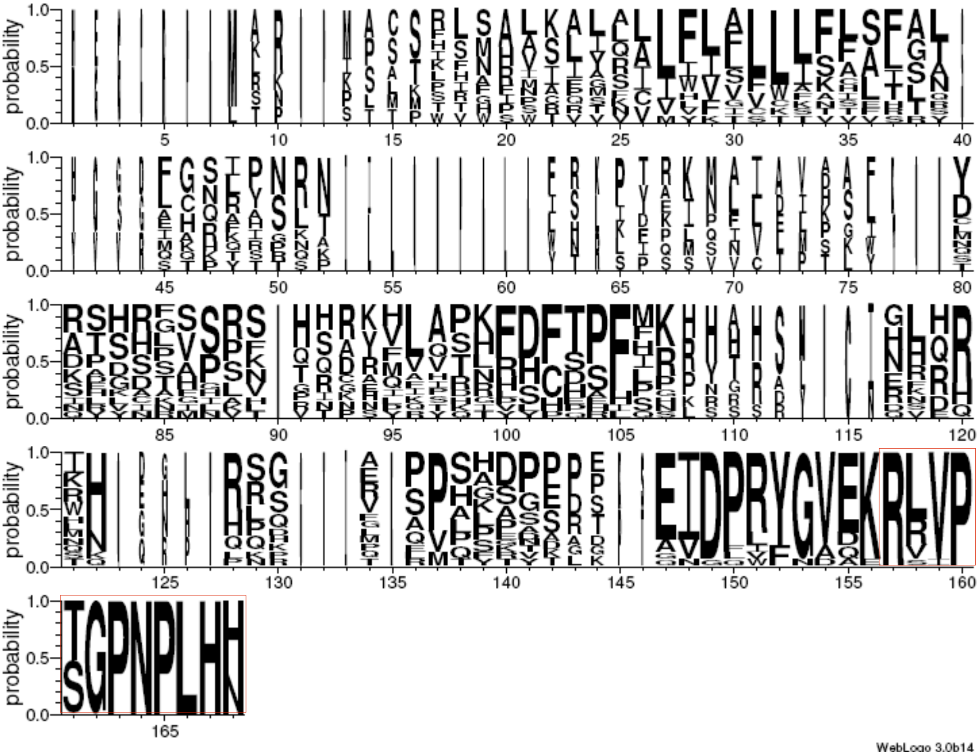

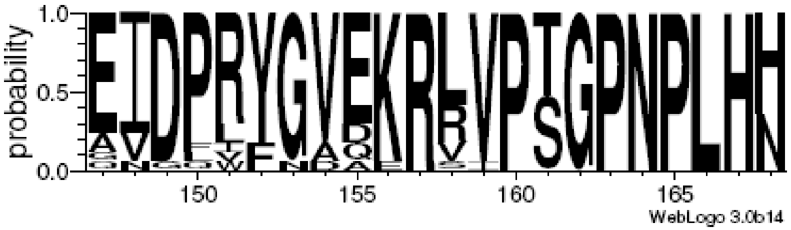

Group10
17 members
dwarf 14/21
AtCLE19
BnCLE19

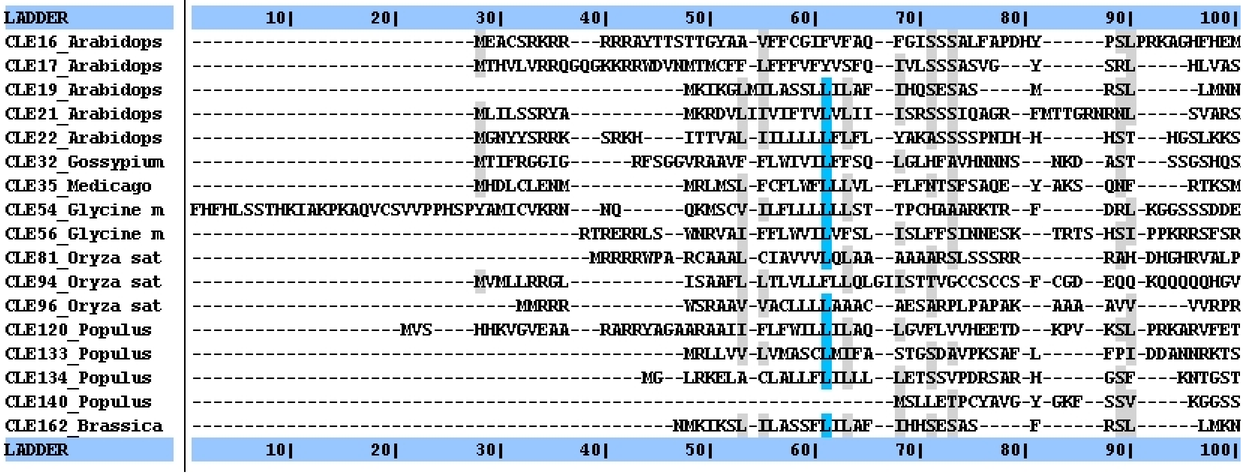

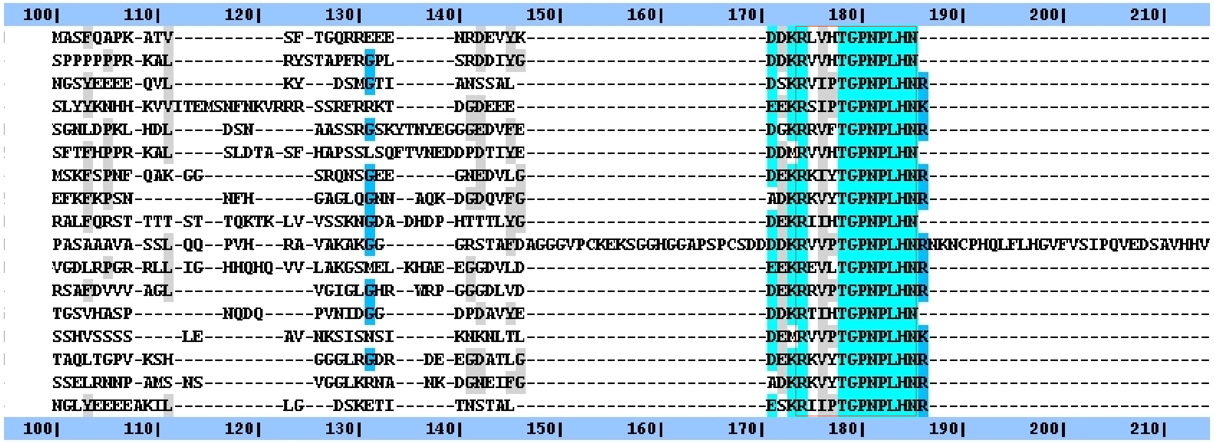

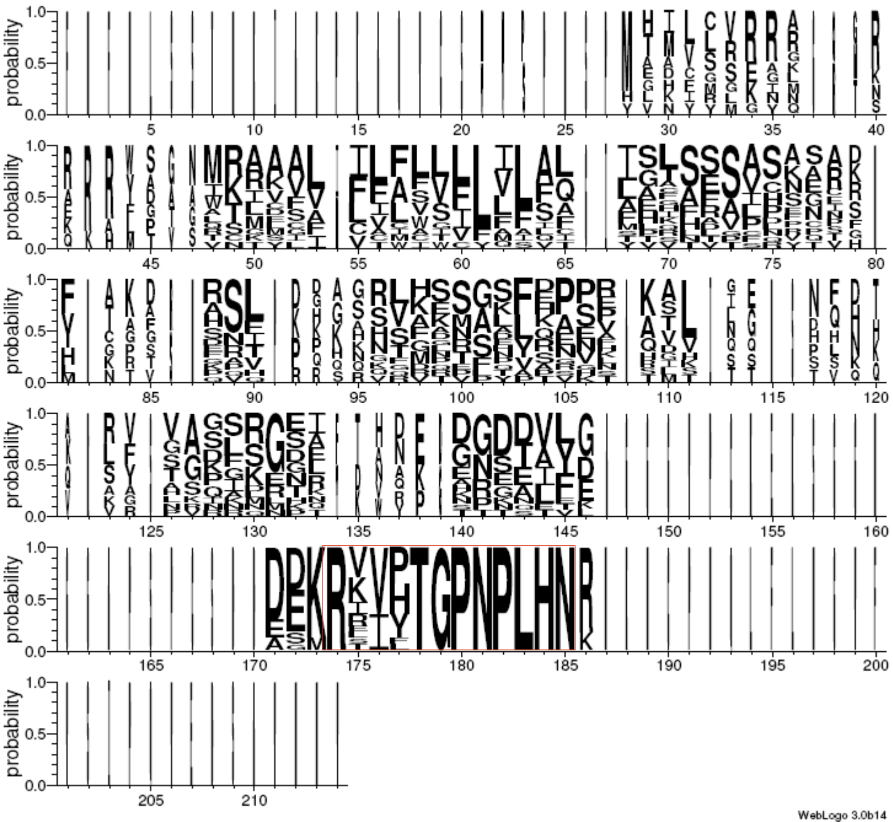

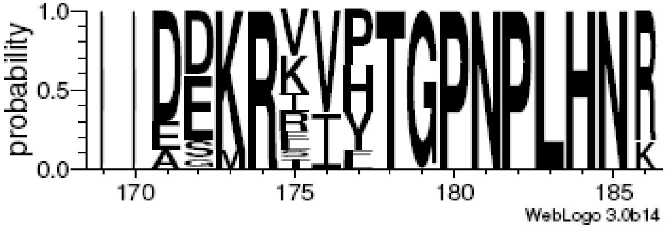

Group11
16 member

```
LADDER                   10|       20|       30|       40|       50|       60|       70|       80|       90|      100|      110|      120|      130|      140|      150|      160|
CLE14_Arabidops ---------------------------------MKVWSQR-LSFLIVMIFILAGLHSSSAGRKL-PSMT------TTEEFQR------LSFDGKRILSEVTA---------------------------DKKYDRIYGASARLVPKGPNPLHNK--------
CLE15_Oryza sat --------------------------------MLRSRKSRVMVMLVTAALLLTDMAGVSYGRRLIPDLD------AMAVVGG------SPPAKGGYMSRLQVP------------------PSDSGHHVGDEYRSMHAVSKRLVPQGPNPLHN---------
CLE37_Medicago  -----------------EQLFSSSNFLVPSIIAMRVLKSS--TILLLLTILLSVMQATTSRNSV-THVT------LVDMERR------SETKLSSMFVHSYSA------------------ILTS---LNNKKGKFHAVSRHVVPSGPNPLHN---------
CLE57_Glycine m -----------------------------------------------------------------RHISWGLKEEIMEQRTR------SRFPFSSFPHSYNSA------------------EIK-----DNKVSGFHTVSHTATPGGPNPLHN---------
CLE58_Glycine m --------------------------LKISIKIMRIPKFS--TLFLLLIVLMSSIQDSDSTSRLESHVE--------LERRL------SERKVSSMFIQSYSA------------------ILSSLNSRKSKFKQIHAVSHRLVPSGPNPLHN---------
CLE63_Glycine m -----------------------------------------------------------------------------------------------------TQ------------------QVPS----TEEVHPIYGVSLRDVPGGPNPLHN---------
CLE92_Oryza sat --------------------------MKPSSSSVGVCTLL-VAVASLQLLLVVAVAASAKTAPAMPDEE-FLGR-LCDQQQG------ATRRRLPWCQQLHARRRHGGGGGGVGVGKRRRVPMPPPSRAGEEIDARYDVSKRVVPSGPNPLHN---------
CLE103_Oryza sa --------------------------QASSIMSSWSWSVR--LMVLTLTLLGAELAGIRHGRRIIPSLE------GDSPSPS------PASKGCGLLTEEMAA------PPGHYYSSRR--MMQ-----EEEEDHGVSASKRVVPEGPNPLHN---------
CLE105_Oryza sa --------------------------------MRRFSKQHLVPFILLLLLVMSHLPISSLGSRR-----------AFREEAV------SGFRSHELAPTMAPS---QEKEAG---------VVAGA---GSICGQKYAVSRRMVPQGPNPLHN---------
CLE128_Populus  ---------------------------------MRIAYSPLLPLLVFSTIMLSLLHPSTC-----RHIS---RA-TYEEEQQ------LNTEFSLPLPQHLPA-----------IAHTV--KFNK----DDKVQKLYAASHKLVPGGPNPLHN---------
CLE129_Populus  --------------------------MGTTTLYPRLS-----FFVLMIMLVVNQLSSCHFIHSR---------T-SEEPGKT------VETDLSSHFSWKFKE------------------MVRERSS-KDESDTIYRVSRRKIPAGPNPLHN---------
CLE130_Populus  ---------------------------------MRIPNPRRSFLFLMIILAISQLSSCRY-----LHIN-I----RDQTNQT------VETDVSTQFSWHFTA------------------KAPEGSNKDEIDDPVYGASYRTVPGGPNPLHN---------
CLE151_Zea mays FELKNHPSSSSSTTTSPSLLFV----LRRSMVRNPLKARP--TLVLISLLLMADLATVGYSRRV-AELG------ALAVSDSGGGGGGSPPGKGCCLPNQSSS------------------SASGRHLGDDCCKQMRIVSKRLVPQGPNPLHN---------
CLE157_Zea mays -----------------------------RPAMRHAPSSP--AMLLLLLLATFTYISCRAPKPD-----------AATAARK-HGGA-VLWTVRPPPRTAPPD------VGG-----------------DMGARAVQGESKRLVPQGPNPLHN---------
CLE159_Zea mays -----------------------------DTLHRRRPCS------------------CCCSWPL-----------SPTSAAE----------LRSPMRRPPPD------VGG-----------------DMGARAVQGESKRLVPQGPNPLHN---------
CLE170_Physcomi -ALVGRSDVEQSW----NVL------VGRRGRFRRLVQQD-DDLVCDAQLTCMKKEVAKSTSSM---LD------VVEGLRT------REILVNYRLPQRKPT------------------PFQ---------------SERMVPSGPNPLHNSNRSQVIFP
LADDER                   10|       20|       30|       40|       50|       60|       70|       80|       90|      100|      110|      120|      130|      140|      150|      160|
```

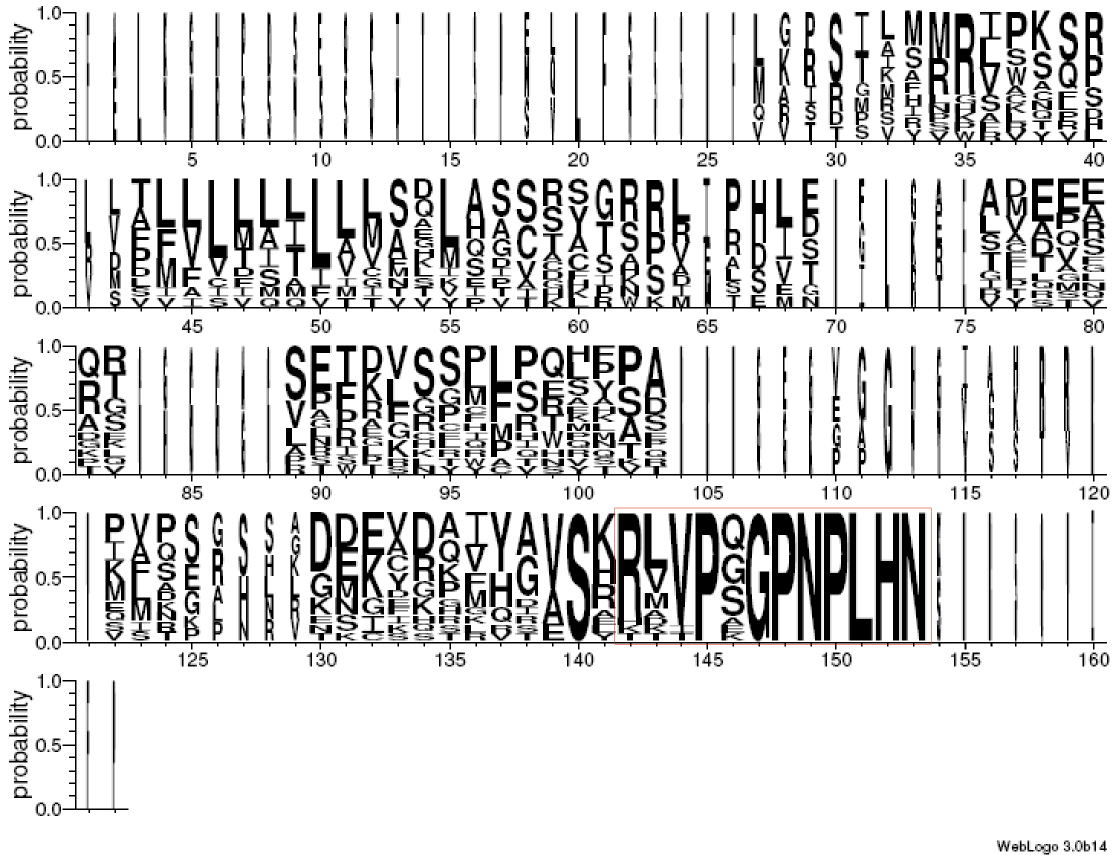

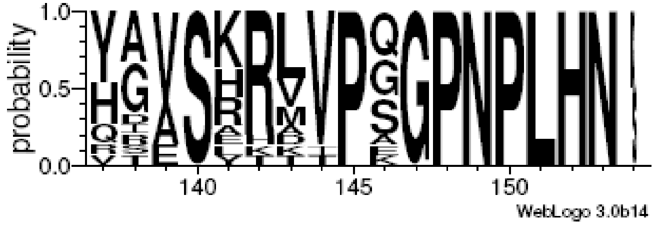

Group12
4 members

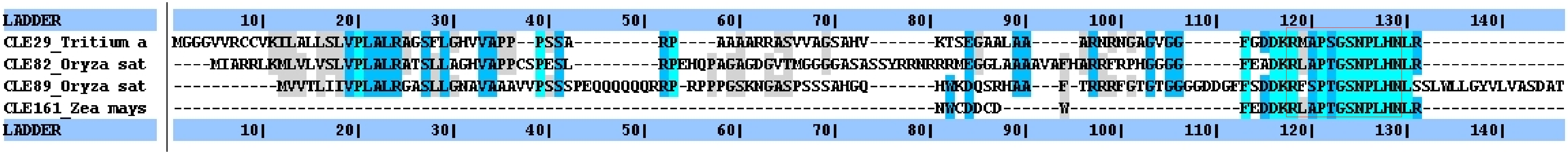

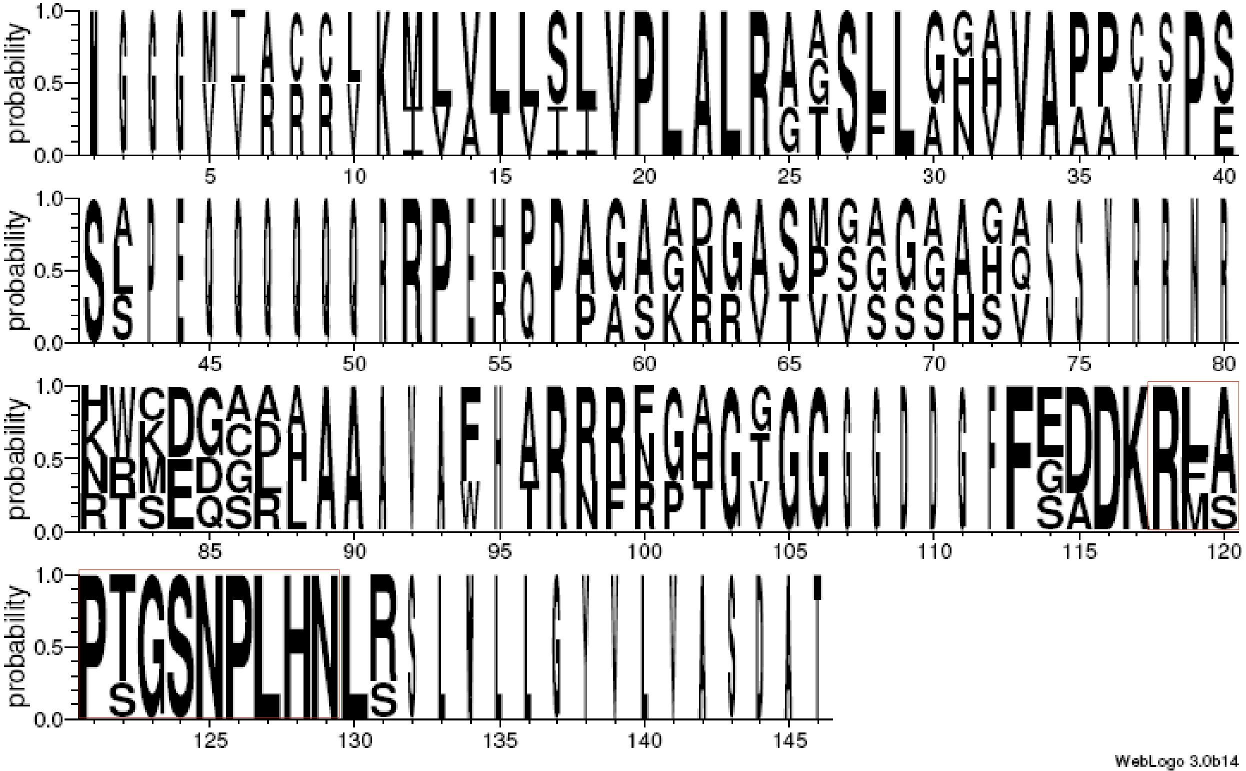

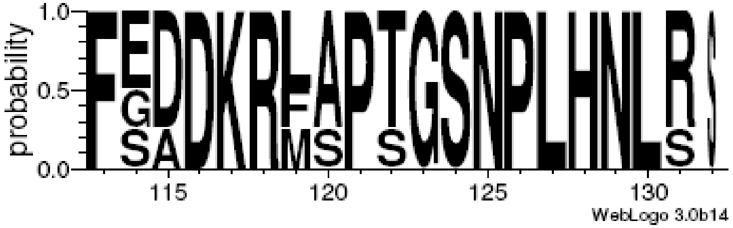

Group13
6 members

```
LADDER                       10|       20|       30|       40|       50|       60|       70|       80|       90|      100|
CLE20_Arabidops    ----------------MKNKNMNPSRPRLLCLIVFLFLVTVLSKASR-IHVERR---RFSSK-PSGENREFL-PSQPTFPV--VDAGEILPDKRKVKTGSNPLHNKR-
CLE23_Glycine m    -----------------TNKTTNMKHFHLFLFLALLFL------TPR-VHAIRI---KFSGP-STSSHQDFH-PWANS-PIRSSREREFMSEKRRVPTGSNPLHNKR-
CLE33_Lycopersi    -----------------------------FFFFIILFT------SPR-GHVIHLQPARFSGPSPRKESVIFQ-PLPTS----------FKFENRRVPTGSNPLHNKKR
CLE70_Medicago     -----------------------MKHFHFFLLLSLLFL------TPR-VYAIRI---KFSSPSSTSSHHAFHIPFFNS-PSR-SKGKEFKSQKRKVPTGSNPLHNKR-
CLE138_Populus     -----------------------MKLFHLFLLLALLIF---FSSTPRSSHAARR---SFSAP--STSQQVFRSPFRAS-PFA-ERAKEFESQKRKVPTGSNPLHNKR-
CLE174_Solanum     IKIIPYLIFQYSQKINILKKV--MNIFHFIFFFLLIIT-----TSSR-IQALRQLPEQVSGMPAARKSQVFH-PIVIA-----PAAQDFESFKRRIPTGTNPLHNKKR
LADDER                       10|       20|       30|       40|       50|       60|       70|       80|       90|      100|
```

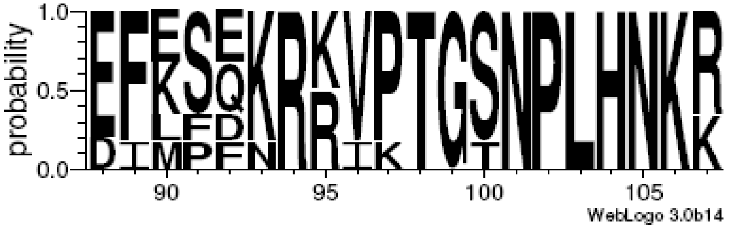

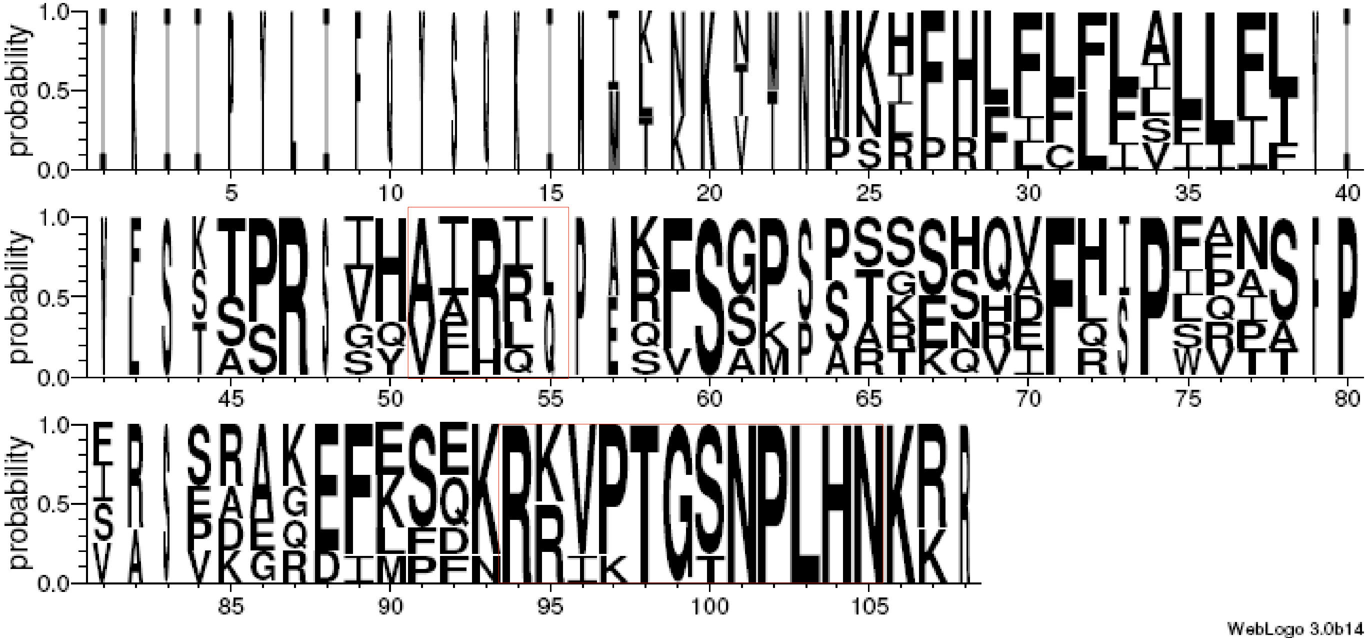

Supplement: Additional file 2 — Multiple sequence alignments of groups and full length sequence logos. [file 1471-2229-8-1-S2.pdf]
