# Supplementary material for: Bioinformatic analysis of the CLE signaling peptide family
Source: BMC Plant Biol. 2009 Feb 9;9:17. doi: 10.1186/1471-2229-9-17 (PMC2645403; doi:10.1186/1471-2229-9-17)
Supplement: Additional file 1 — Full listing of CLE peptide numbers for each group of CLE peptides, sorted by CLE peptide number. Table: The groups correspond to those shown in Figures 2 and 3 of the original article [1]. [file 1471-2229-9-17-S1.doc]

**Table 2:** Full listing of CLE peptide numbers for each group of CLE peptides, sorted by CLE peptide number

| **CLE number** | **Group** | **Comment** |
| --- | --- | --- |
| CLE1 | Group2 |  |
| CLE2 | Group2 |  |
| CLE3 | Group2 |  |
| CLE4 | Group2 |  |
| CLE5 | Group2 |  |
| CLE6 | Group2 |  |
| CLE7 | Group2 |  |
| CLE8 | no_group |  |
| CLE9 | Group9 |  |
| CLE10 | Group9 |  |
| CLE11 | Group9 |  |
| CLE12 | Group9 |  |
| CLE13 | Group9 |  |
| CLE14 | Group11 |  |
| CLE15 | Group11 |  |
| CLE16 | Group10 |  |
| CLE17 | Group10 |  |
| CLE18 | no_group |  |
| CLE19 | Group10 |  |
| CLE20 | Group13 |  |
| CLE21 | Group10 |  |
| CLE22 | Group10 |  |
| CLE23 | Group13 |  |
| CLE24 | Group8 |  |
| CLE25 | Group7 |  |
| CLE26 | Group7 |  |
| CLE27 | Group8 |  |
| CLE28 | No group |  |
| CLE29 | Group12 |  |
| CLE30 | not included, multi CLE |  |
| CLE31 | not included, multi CLE |  |
| CLE32 | Group10 |  |
| CLE33 | Group13 |  |
| CLE34 | Group7 |  |
| CLE35 | no_group | Corrected |
| CLE36 | Group7 | Corrected |
| CLE37 | no_group | Corrected |
| CLE38 | no_group | Corrected |
| CLE39 | Group11 | Corrected |
| CLE40 | Group4 |  |
| CLE41 | Group5 |  |
| CLE42 | Group5 |  |
| CLE43 | no_group |  |
| CLE44 | Group5 |  |
| CLE45 | Group6 |  |
| CLE46 | Group3 |  |
| CLE47 | Group2 |  |
| CLE48 | Group3 |  |
| CLE49 | Group5 |  |
| CLE50 | no_group |  |
| CLE51 | Group5 |  |
| CLE52 | Group9 |  |
| CLE53 | Group5 |  |
| CLE54 | Group10 |  |
| CLE55 | Group8 |  |
| CLE56 | Group10 |  |
| CLE57 | Group11 |  |
| CLE58 | Group11 |  |
| CLE59 | no_group |  |
| CLE60 | Group5 |  |
| CLE61 | Group4 |  |
| CLE62 | no_group |  |
| CLE63 | Group11 |  |
| CLE64 | no_group | Corrected |
| CLE65 | Group5 | Corrected |
| CLE66 | Group9 | Corrected |
| CLE67 | Group8 | Corrected |
| CLE68 | not included, multi CLE | Corrected |
| CLE69 | Group9 | Corrected |
| CLE70 | Group9 | Corrected |
| CLE71 | Group10 | Corrected |
| CLE72 | no_group | Corrected |
| CLE73 | Group13 | Corrected |
| CLE74 | no_group | Corrected |
| CLE75 | not included, multi CLE |  |
| CLE76 | not included, multi CLE |  |
| CLE77 | Group8 |  |
| CLE78 | Group7 |  |
| CLE79 | Group6 |  |
| CLE80 | Group7 |  |
| CLE81 | Group10 |  |
| CLE82 | Group12 |  |
| CLE83 | Group9 |  |
| CLE84 | no_group |  |
| CLE85 | Group7 |  |
| CLE86 | Group7 |  |
| CLE87 | no_group |  |
| CLE88 | no_group |  |
| CLE89 | Group12 |  |
| CLE90 | Group5 |  |
| CLE91 | Group9 |  |
| CLE92 | Group11 |  |
| CLE93 | Group4 |  |
| CLE94 | Group10 |  |
| CLE95 | Group2 |  |
| CLE96 | Group10 |  |
| CLE97 | no_group |  |
| CLE98 | Group6 |  |
| CLE99 | Group2 |  |
| CLE100 | Group6 |  |
| CLE101 | no_group |  |
| CLE102 | Group2 |  |
| CLE103 | Group11 |  |
| CLE104 | Group2 |  |
| CLE105 | Group11 |  |
| CLE106 | Group1 |  |
| CLE107 | no_group |  |
| CLE108 | Group1 |  |
| CLE109 | no_group |  |
| CLE110 | Group2 |  |
| CLE111 | no_group |  |
| CLE112 | no_group |  |
| CLE113 | no_group |  |
| CLE114 | no_group |  |
| CLE115 | no_group |  |
| CLE116 | Group5 |  |
| CLE117 | Group7 |  |
| CLE118 | Group7 |  |
| CLE119 | Group5 |  |
| CLE120 | Group10 |  |
| CLE121 | no_group |  |
| CLE122 | Group9 |  |
| CLE123 | Group9 |  |
| CLE124 | Group4 |  |
| CLE125 | Group3 |  |
| CLE126 | Group7 |  |
| CLE127 | no_group |  |
| CLE128 | Group11 |  |
| CLE129 | Group11 |  |
| CLE130 | Group11 |  |
| CLE131 | no_group |  |
| CLE132 | Group2 |  |
| CLE133 | Group10 |  |
| CLE134 | Group10 |  |
| CLE135 | Group2 |  |
| CLE136 | Group2 |  |
| CLE137 | Group2 |  |
| CLE138 | Group13 |  |
| CLE139 | Group2 |  |
| CLE140 | Group10 |  |
| CLE141 | Group9 |  |
| CLE142 | Group9 |  |
| CLE143 | Group3 |  |
| CLE144 | ZmESR |  |
| CLE145 | ZmESR |  |
| CLE146 | ZmESR |  |
| CLE147 | Group3 |  |
| CLE148 | Group5 |  |
| CLE149 | Group5 |  |
| CLE150 | Group4 |  |
| CLE151 | Group11 |  |
| CLE152 | Group1 |  |
| CLE153 | no_group |  |
| CLE154 | no_group |  |
| CLE155 | no_group |  |
| CLE156 | no_group |  |
| CLE157 | Group11 |  |
| CLE158 | no_group |  |
| CLE159 | Group11 |  |
| CLE160 | no_group |  |
| CLE161 | Group12 |  |
| CLE162 | Group10 |  |
| CLE163 | Group7 |  |
| CLE164 | Group7 |  |
| CLE165 | Group5 |  |
| CLE166 | Group5 |  |
| CLE167 | Group5 |  |
| CLE168 | Group7 |  |
| CLE169 | Group5 |  |
| CLE170 | Group11 |  |
| CLE171 | no_group |  |
| CLE172 | Group5 |  |
| CLE173 | Group9 |  |
| CLE174 | Group13 |  |
| CLE175 | Group9 |  |
| CLE176 | Group8 |  |
| CLE177 | no_group |  |
| CLE178 | no_group |  |
| CLE179 | no_group |  |

The groups correspond to those shown in Figures 2 and 3 of the original article [1].
