# Supplementary material for: Bioinformatic analysis of the CLE signaling peptide family
Source: BMC Plant Biol. 2009 Feb 9;9:17. doi: 10.1186/1471-2229-9-17 (PMC2645403; doi:10.1186/1471-2229-9-17)
Supplement: Additional file 2 — Full listing of CLE peptide numbers for each group of CLE peptides, sorted by CLE peptide groups. Table: The groups correspond to those shown in Figures 2 and 3 of the original article [1]. [file 1471-2229-9-17-S2.doc]

**Table 3:** Full listing of CLE peptide numbers for each group of CLE peptides, sorted by CLE peptide groups

| **Group** | **CLE number** | **Comment** |
| --- | --- | --- |
| Group1 | CLE106 |  |
| Group1 | CLE108 |  |
| Group1 | CLE152 |  |
| Group2 | CLE1 |  |
| Group2 | CLE2 |  |
| Group2 | CLE3 |  |
| Group2 | CLE4 |  |
| Group2 | CLE5 |  |
| Group2 | CLE6 |  |
| Group2 | CLE7 |  |
| Group2 | CLE47 |  |
| Group2 | CLE95 |  |
| Group2 | CLE99 |  |
| Group2 | CLE102 |  |
| Group2 | CLE104 |  |
| Group2 | CLE110 |  |
| Group2 | CLE132 |  |
| Group2 | CLE135 |  |
| Group2 | CLE136 |  |
| Group2 | CLE137 |  |
| Group2 | CLE139 |  |
| Group3 | CLE46 |  |
| Group3 | CLE48 |  |
| Group3 | CLE125 |  |
| Group3 | CLE143 |  |
| Group3 | CLE147 |  |
| Group4 | CLE40 |  |
| Group4 | CLE61 |  |
| Group4 | CLE93 |  |
| Group4 | CLE124 |  |
| Group4 | CLE150 |  |
| Group5 | CLE65 | corrected |
| Group5 | CLE41 |  |
| Group5 | CLE42 |  |
| Group5 | CLE44 |  |
| Group5 | CLE49 |  |
| Group5 | CLE51 |  |
| Group5 | CLE53 |  |
| Group5 | CLE60 |  |
| Group5 | CLE90 |  |
| Group5 | CLE116 |  |
| Group5 | CLE119 |  |
| Group5 | CLE148 |  |
| Group5 | CLE149 |  |
| Group5 | CLE165 |  |
| Group5 | CLE166 |  |
| Group5 | CLE167 |  |
| Group5 | CLE169 |  |
| Group5 | CLE172 |  |
| Group6 | CLE45 |  |
| Group6 | CLE79 |  |
| Group6 | CLE98 |  |
| Group6 | CLE100 |  |
| Group7 | CLE25 |  |
| Group7 | CLE26 |  |
| Group7 | CLE34 |  |
| Group7 | CLE36 | corrected |
| Group7 | CLE78 |  |
| Group7 | CLE80 |  |
| Group7 | CLE85 |  |
| Group7 | CLE86 |  |
| Group7 | CLE117 |  |
| Group7 | CLE118 |  |
| Group7 | CLE126 |  |
| Group7 | CLE163 |  |
| Group7 | CLE164 |  |
| Group7 | CLE168 |  |
| Group8 | CLE24 |  |
| Group8 | CLE27 |  |
| Group8 | CLE55 |  |
| Group8 | CLE67 | corrected |
| Group8 | CLE77 |  |
| Group8 | CLE176 |  |
| Group9 | CLE9 |  |
| Group9 | CLE10 |  |
| Group9 | CLE11 |  |
| Group9 | CLE12 |  |
| Group9 | CLE13 |  |
| Group9 | CLE70 | corrected |
| Group9 | CLE52 |  |
| Group9 | CLE69 | corrected |
| Group9 | CLE66 | corrected |
| Group9 | CLE83 |  |
| Group9 | CLE91 |  |
| Group9 | CLE122 |  |
| Group9 | CLE123 |  |
| Group9 | CLE141 |  |
| Group9 | CLE142 |  |
| Group9 | CLE173 |  |
| Group9 | CLE175 |  |
| ZmESR | CLE144 |  |
| ZmESR | CLE145 |  |
| ZmESR | CLE146 |  |
| Group10 | CLE16 |  |
| Group10 | CLE17 |  |
| Group10 | CLE19 |  |
| Group10 | CLE21 |  |
| Group10 | CLE22 |  |
| Group10 | CLE32 |  |
| Group10 | CLE71 | corrected |
| Group10 | CLE54 |  |
| Group10 | CLE56 |  |
| Group10 | CLE81 |  |
| Group10 | CLE94 |  |
| Group10 | CLE96 |  |
| Group10 | CLE120 |  |
| Group10 | CLE133 |  |
| Group10 | CLE134 |  |
| Group10 | CLE140 |  |
| Group10 | CLE162 |  |
| Group11 | CLE14 |  |
| Group11 | CLE15 |  |
| Group11 | CLE39 | corrected |
| Group11 | CLE57 |  |
| Group11 | CLE58 |  |
| Group11 | CLE63 |  |
| Group11 | CLE92 |  |
| Group11 | CLE103 |  |
| Group11 | CLE105 |  |
| Group11 | CLE128 |  |
| Group11 | CLE129 |  |
| Group11 | CLE130 |  |
| Group11 | CLE151 |  |
| Group11 | CLE157 |  |
| Group11 | CLE159 |  |
| Group11 | CLE170 |  |
| Group12 | CLE29 |  |
| Group12 | CLE82 |  |
| Group12 | CLE89 |  |
| Group12 | CLE161 |  |
| Group13 | CLE20 |  |
| Group13 | CLE23 |  |
| Group13 | CLE33 |  |
| Group13 | CLE73 | corrected |
| Group13 | CLE138 |  |
| Group13 | CLE174 |  |
| not included, multi CLE | CLE30 |  |
| not included, multi CLE | CLE31 |  |
| not included, multi CLE | CLE68 |  |
| not included, multi CLE | CLE75 |  |
| not included, multi CLE | CLE76 |  |
| no_group | CLE8 |  |
| no_group | CLE18 |  |
| no_group | CLE28 |  |
| no_group | CLE74 | corrected |
| no_group | CLE43 |  |
| no_group | CLE50 |  |
| no_group | CLE59 |  |
| no_group | CLE62 |  |
| no_group | CLE64 | corrected |
| no_group | CLE35 | corrected |
| no_group | CLE38 | corrected |
| no_group | CLE72 | corrected |
| no_group | CLE37 | corrected |
| no_group | CLE84 |  |
| no_group | CLE87 |  |
| no_group | CLE88 |  |
| no_group | CLE97 |  |
| no_group | CLE101 |  |
| no_group | CLE107 |  |
| no_group | CLE109 |  |
| no_group | CLE111 |  |
| no_group | CLE112 |  |
| no_group | CLE113 |  |
| no_group | CLE114 |  |
| no_group | CLE115 |  |
| no_group | CLE121 |  |
| no_group | CLE127 |  |
| no_group | CLE131 |  |
| no_group | CLE153 |  |
| no_group | CLE154 |  |
| no_group | CLE155 |  |
| no_group | CLE156 |  |
| no_group | CLE158 |  |
| no_group | CLE160 |  |
| no_group | CLE171 |  |
| no_group | CLE177 |  |
| no_group | CLE178 |  |
| no_group | CLE179 |  |

The groups correspond to those shown in Figures 2 and 3 of the original article [1].
